# An improved trajectory tracking control of quadcopter using a novel Sliding Mode Control with Fuzzy PID Surface

**DOI:** 10.1371/journal.pone.0308997

**Published:** 2024-11-21

**Authors:** Elisabeth Andarge Gedefaw, Chala Merga Abdissa, Lebsework Negash Lemma

**Affiliations:** School of Electrical and Computer Engineering, Addis Ababa University, Addis Ababa, Ethiopia; University of Hull, UNITED KINGDOM OF GREAT BRITAIN AND NORTHERN IRELAND

## Abstract

This paper presents Super Twisting Sliding Mode Control with a novel Fuzzy PID Surface for improved trajectory tracking of quadrotor unmanned aerial vehicles under external disturbances. First, quadrotor dynamic model with six degrees of freedom (6-DOF) is developed using Newton-Euler Method. Then, a robust Sliding Mode Control based on a new Fuzzy PID Surface is designed to be capable of automatically adjusting its gain parameters. The proposed SMC controller applies super twisting algorithm with PID surface to reduce chattering and a fuzzy logic controller to automatically adjust the gain parameters in order to enhance robustness. Furthermore, the solution to stability has been given by the Lyapunov method. The controller’s performance is tested through various trajectories, parameter variations, and disturbance scenarios, comparing it with recent alternatives such as Sliding Mode Control, Fuzzy Sliding Mode Control, and Fuzzy Super Twisting Sliding Mode Control using numerical simulations. The simulation results show that the proposed controller has better tracking performance, parameter variation handling, and disturbance rejection capability compared with the aforementioned controllers. Additionally, the control efforts of the proposed method are minimal and smooth, proving it to be an economically feasible controller and operationally safe for the quadrotor.

## 1 Introduction

Unmanned aerial vehicles have evolved into highly advanced robotic systems employed for diverse tasks, including agricultural spraying, power line fault detection, and traffic monitoring [1–5]. These UAVs fall into two main categories: fixed-wing and multi-copter (rotary-wing) aircraft. Fixed-wing UAVs, which employ wings similar to traditional airplanes, are highly efficient for forward motion but lack the mobility crucial to multi-copter UAVs. Multi-copter UAVs, characterized by three or more propellers, offer maneuverability and the ability to hover over targets. Among multi-copters, the quadcopter, featuring four motor-driven rotors on a fixed frame, is the most widely used [6–9].

A quadcopter, with its six degrees of freedom (DOF), is capable of three directional translations (x, y, z) and rotation (roll, pitch, yaw) around three axes. The control of quadcopters poses a significant challenge due to their highly nonlinear dynamics. Therefore, robust controllers are essential, with sliding mode controllers being one such category [10–13].

Backstepping control is one approach used to manage the nonlinear dynamics of quadcopters. In [14], backstepping and integral backstepping methods for the attitude and altitude control of a quadrotor are utilized, significantly improving precision and robustness while increasing the control system’s complexity. In [15], a full control approach for a quadrotor is implemented, combining PD control with sliding mode techniques, achieving good performance in terms of stability and response time, albeit with increased tuning effort. Additionally, adaptive backstepping has been employed for fault-tolerant control, as shown [16], where it is used for fault estimation and reconfiguration of the controller, demonstrating enhanced tracking performance and robustness in the presence of faults. However, backstepping control can introduce complexity in the controller design and require extensive tuning, particularly when dealing with highly nonlinear systems and faults, which may limit its practical implementation.

Sliding mode control is another suitable algorithm for addressing the challenges posed by intricate high-order systems operating in uncertain conditions. It possesses several advantageous features, including the ability to reduce system order and reject disturbances effectively [17–21]. However, it suffers from a drawback known as chattering [22–25]. Chattering manifests as a rapid, high-frequency switching motion between two control laws, occurring in the nearby area of the sliding surface. In an effort to keep the state trajectory on this surface, the switching functions switch at a high frequency. Regrettably, this unwanted chattering effect generates heat due to the rapid and continuous transitions between the switching functions when the control is applied to electronic or mechanical systems. This heat can potentially lead to electronic systems overheating and sustaining damage, while mechanical components can wear out prematurely and suffer eventual damage as a consequence of the abrupt shifts between the control laws.

Various strategies have been employed to mitigate chattering, including the use of saturation functions instead of discontinuous switching functions and the application of higher-order SMC methods [26–28]. However, the former reduces resilience to disturbances, and the latter presents challenges in gain tuning [29, 30]. The neuro-adaptive arbitrary order sliding mode control strategy outlined in [31] integrates high gain differentiation to further address chattering, offering a refined approach that balances robustness and control precision. However, this method also presents its own drawbacks, such as increased computational demands and the complexity associated with neural network training and maintenance. These factors can complicate real-time application and scalability, particularly in systems where hardware capabilities are limited or rapid response is critical.

When dealing with matched perturbations, the high-order sliding mode control method can be employed to drive the sliding variable and its subsequent derivatives to zero. Nonetheless, a significant limitation of high-order SMC is its dependence on data from the high-order time derivatives of the sliding variable [32–34]. Among the higher-order sliding mode control methods, it’s essential to emphasize that second-order SMC, such as the super-twisting algorithm, exclusively requires feedback from the sliding surface during the control process [35].

The super twisting sliding mode controller is a specific approach within sliding mode control used for controlling dynamic systems. It alleviates the conventional sliding mode control by introducing a super twisting algorithm. This algorithm enables rapid and finite-time convergence to the sliding surface, even in the presence of disturbances. It accomplishes this by utilizing a continuous control law that minimizes chattering [36–38]. It’s important to note that the effectiveness of the super twisting sliding mode controller relies on knowledge of perturbation bounds. In practical scenarios, drones face disturbances that can diminish control efficiency, necessitating online tuning of controller parameters.

An adaptive sliding mode control has been designed, featuring automatic adjustments in switching gains to accommodate disturbances [39, 40]. While the system effectively ensures robust trajectory tracking, it does not address the mitigation of chattering. The works [41–43] have facilitated practical implementation; however, it is noteworthy that these contributions have led to an escalation in complexity within the domain.

In [44], an adaptive sliding mode control based on neural networks for nonlinear systems is proposed. This approach dynamically adapts to system uncertainties and disturbances, though it introduces complexity in neural network training and computational burden, potentially limiting real-time applications. In [45], an adaptive sliding mode control for a class of nonlinear systems is developed, which improves control precision and robustness, especially in the presence of parameter variations. However, it requires accurate system modeling, which is not always feasible in practical scenarios. In [46], an adaptive FIT-SMC approach is proposed, incorporating a robust exact differentiator and neural network-based friction compensation, enhancing control precision but increasing computational complexity.

In [47], the authors explore bioinspired machine learning algorithms for optimal path planning in faulty unmanned air vehicles. The study aims to minimize both distance and time, demonstrating enhanced robustness and efficiency, though increased computational complexity limits real-time applicability.

A Fuzzy-PI controller was created to regulate the quadcopter’s position and orientation [48]. This controller automatically adjusts its control parameters, but it exhibits a persistent steady-state error. In [49–51], a fuzzy adaptive sliding mode control for nonlinear systems is introduced. This approach combines the robustness of SMC with the adaptability of fuzzy logic, enhancing control performance while increasing the complexity of the control algorithm and computational demands, potentially hindering its application in systems with limited hardware resources.

Furthermore, several studies have focused on the application of these control strategies to quadcopters. In [52], the real-time stabilization and tracking of a four-rotor mini-rotorcraft using nonlinear control is investigated. This method ensures stability and robustness but demands high computational resources.

Inspired by prior research in the field, this paper suggests applying a super twisting SMC with a Fuzzy PID surface to tune parameters of the controller online, reduce chattering, and steady state error.

The primary contribution of this paper is summarized as follows:

- The integration of an integral term into the sliding surface systematically reduces steady-state error. This enhancement significantly elevates the precision and reliability of the control system, ensuring exact adherence to the desired operational state without the presence of persistent deviations.
- A super twisting sliding mode controller with a PID surface is utilized to reduce chattering and control effort, thereby improving both the operational smoothness and efficiency of the system.
- The parameters of the PID surface are automatically adjusted by employing a fuzzy logic controller, enhancing robustness and disturbance rejection capacity. Through this strategic adjustment, a fast response to varying conditions is enabled, significantly enhancing the system’s resilience and precision in control.

The remaining sections of the manuscript are structured as follows. Section 2 provides a brief presentation of the quadcopter’s kinematic and dynamic equations. Section 3 focuses on the proposed Fuzzy Super Twisting SMC with PID surface designed for the trajectory control of the quadcopter’s attitude and position, respectively. Evaluation of the proposed controllers, along with simulation results and discussions, is presented in Section 4. Finally, Section 5 concludes the paper with a summary and outlines potential future work.

## 2 Quadcopter dynamics

### 2.1 System description

**Fig 1:**
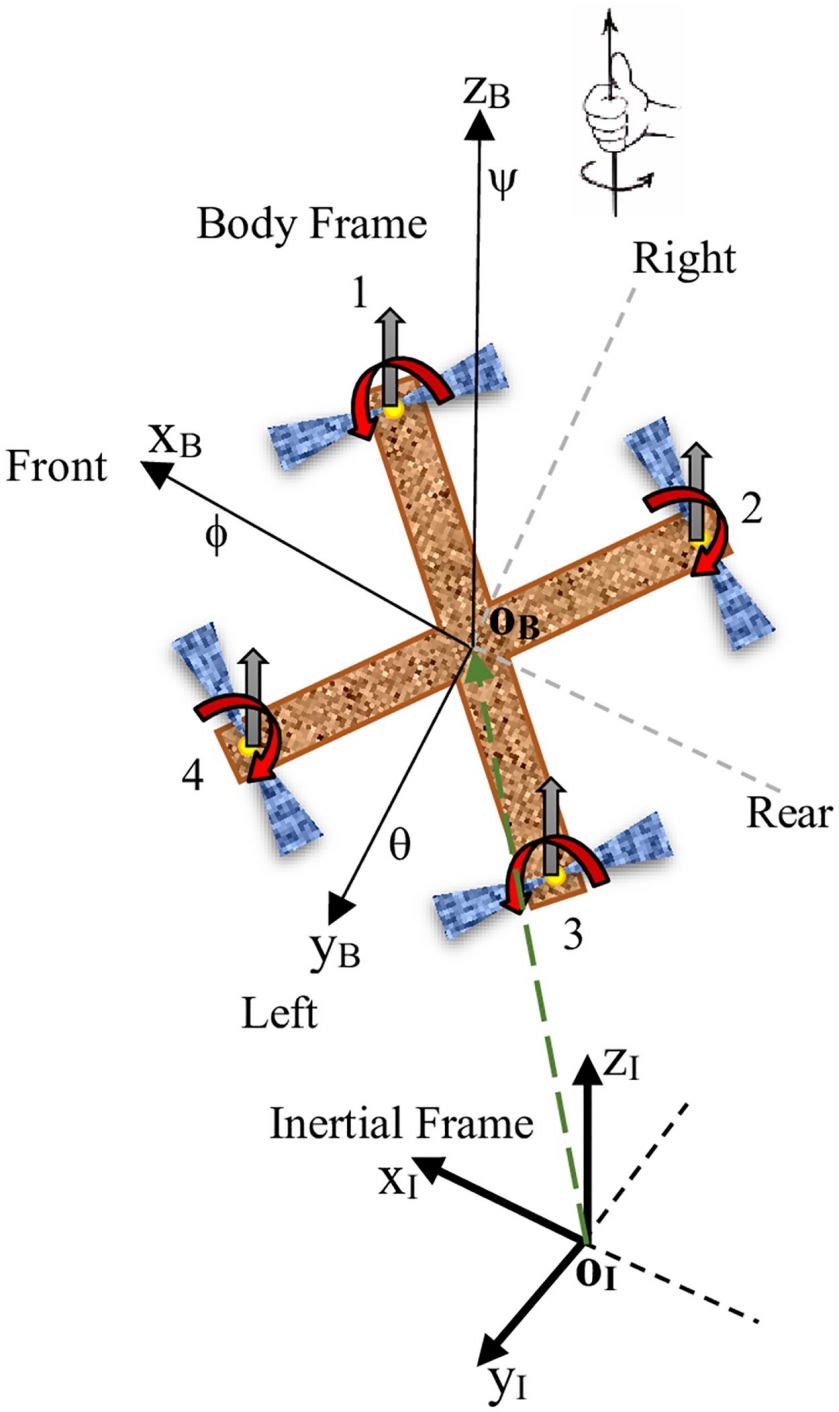
Quadcopter configuration.

The mechanical structure of the quadcopter consists of four uniform arms that are evenly spaced apart [53, 54]. At each end of these arms, there is a motor connected to a propeller through a direct coupling mechanism as illustrated in Fig 1. The propellers are grouped in pairs, with each pair rotating in opposite directions. The motor and propeller system together generate both thrust and torque.

Attaining the six degrees of freedom in a quadcopter is achieved by adjusting the speed of its four motors.

**Fig 2:**
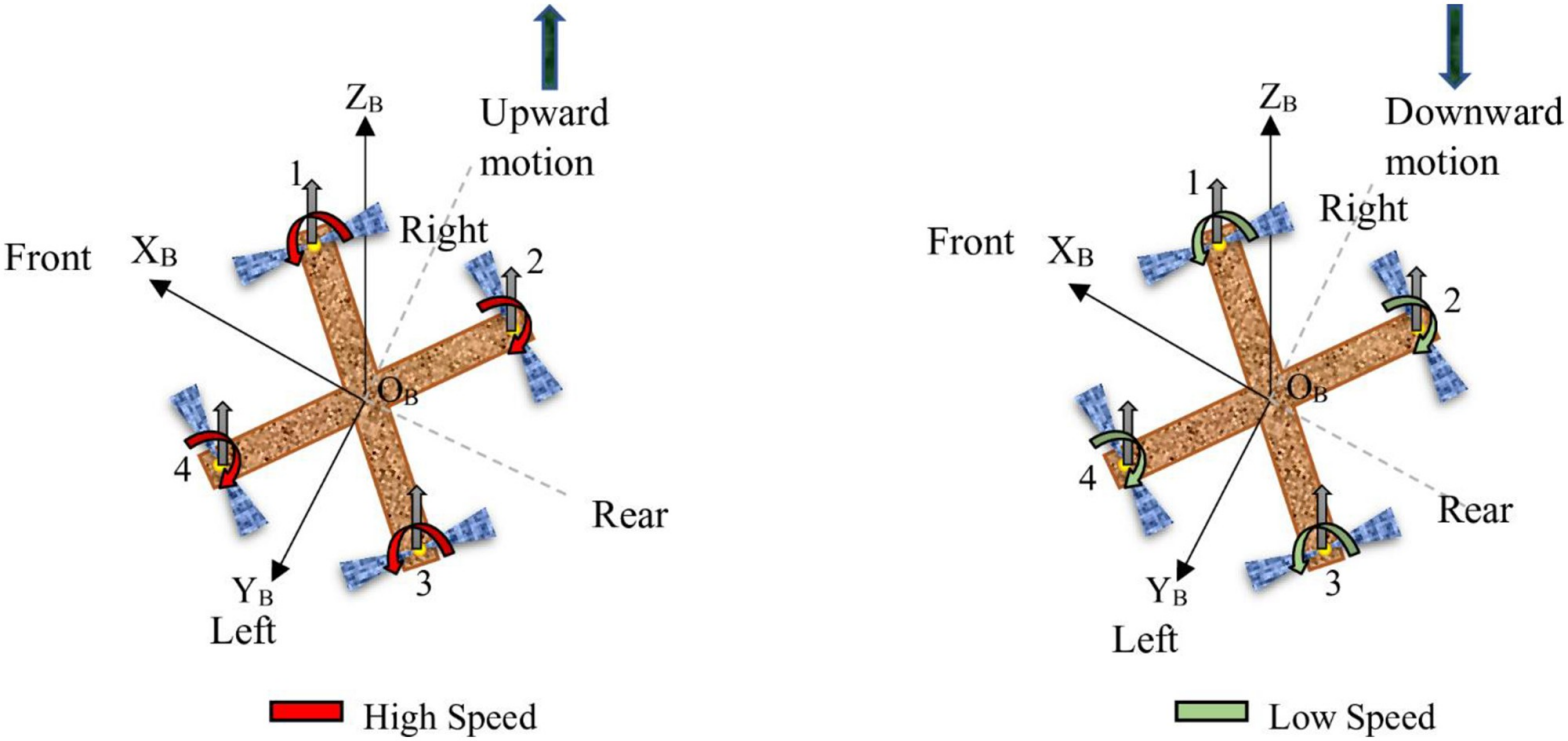
Throttle.

**Fig 3:**
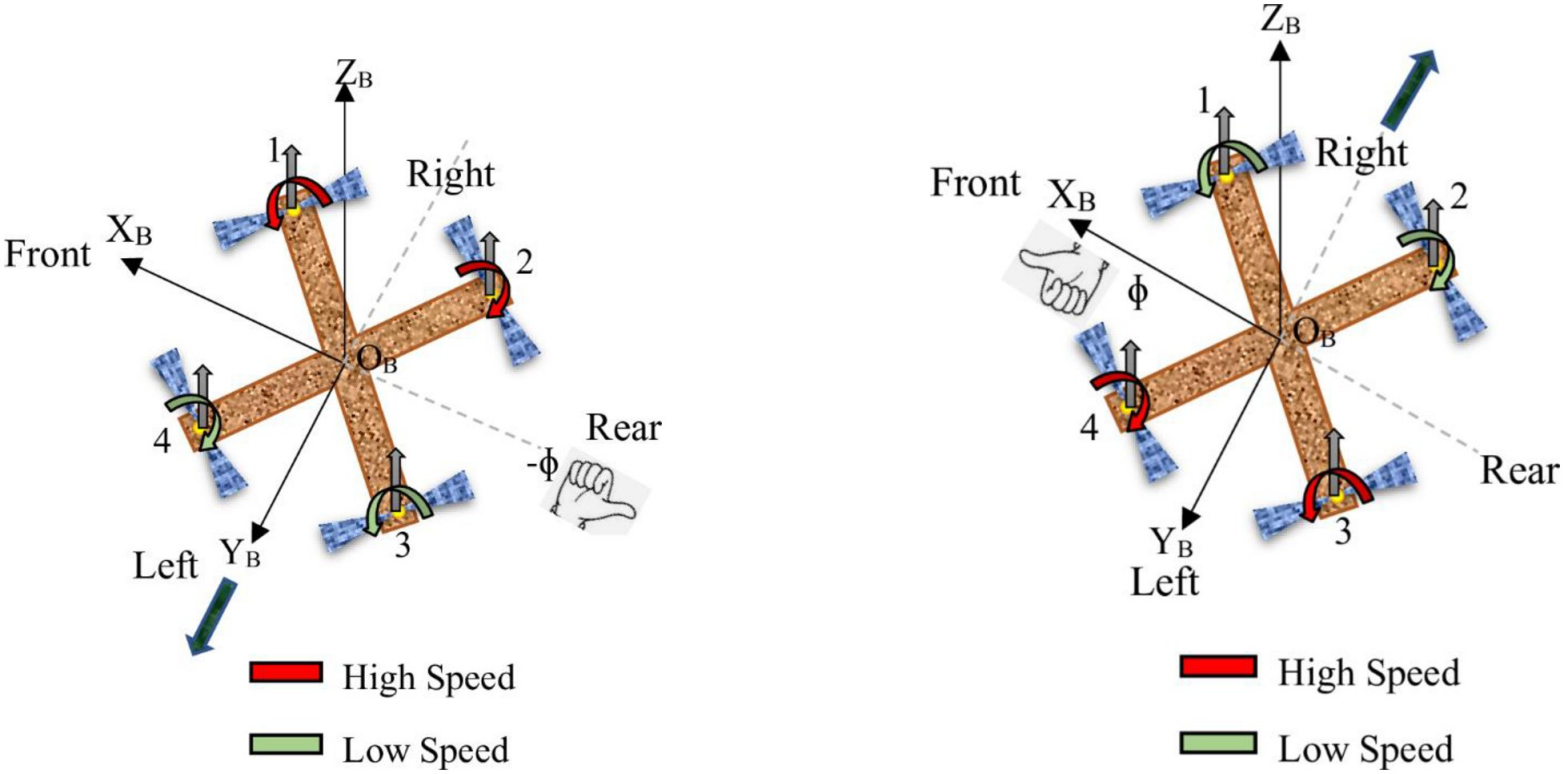
Roll.

**Fig 4:**
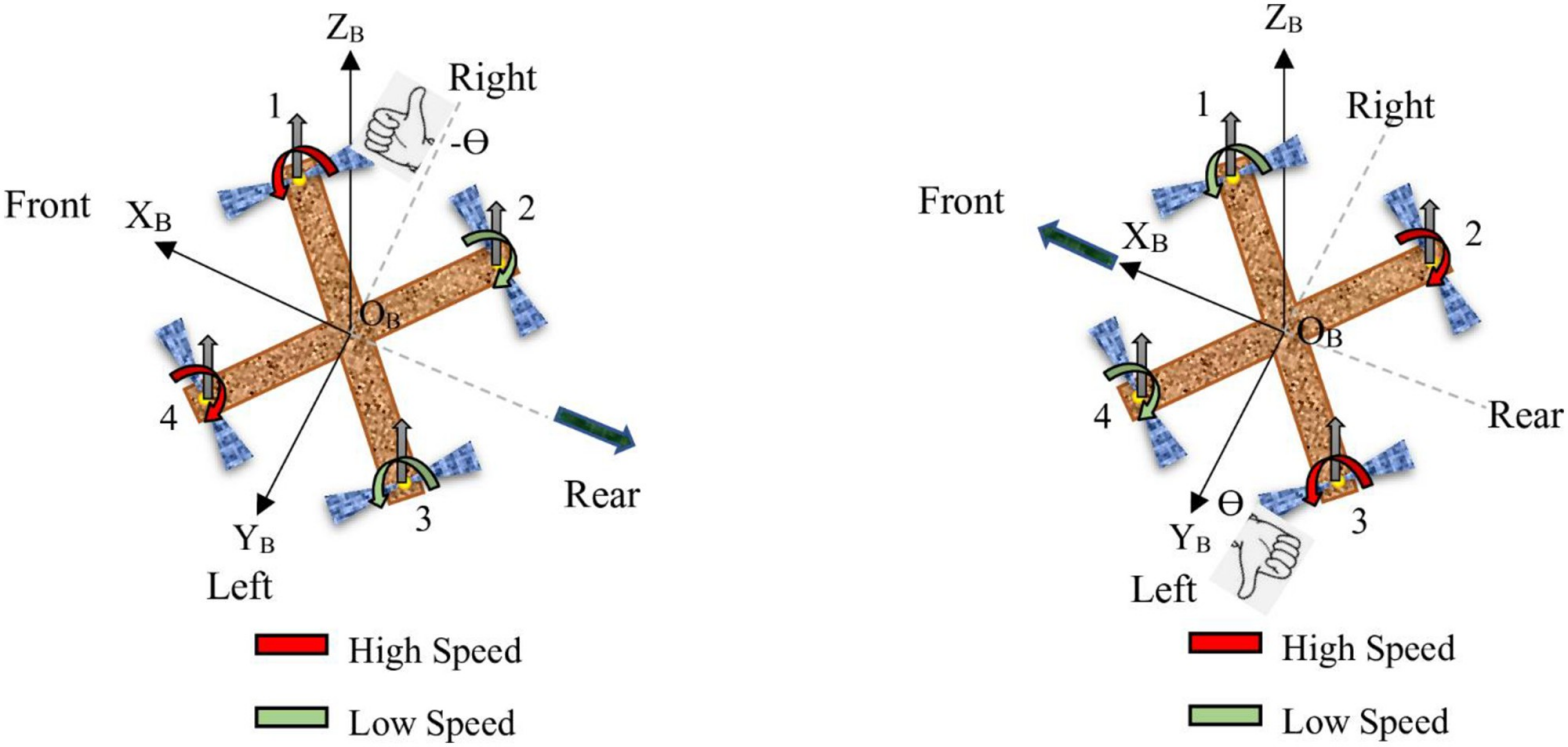
Pitch.

**Fig 5:**
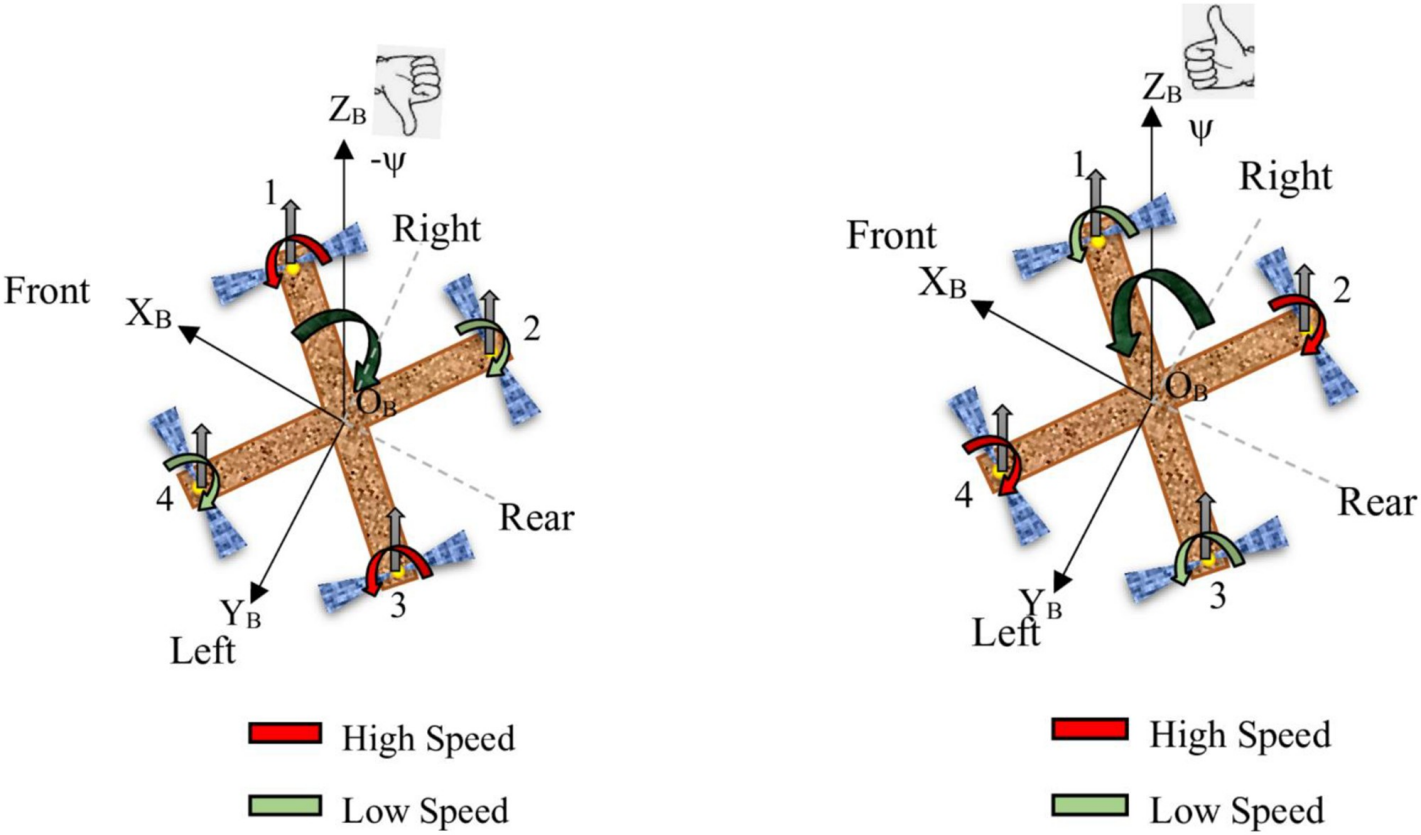
Yaw.

- **Throttle:** Achieving vertical take-off and landing in a quadcopter is made possible through throttling, a process where all propeller speeds *ω*_1_, *ω*_2_, *ω*_3_, and *ω*_4_ are simultaneously set to equal values. This simultaneous adjustment of propeller speeds generates vertical thrust relative to the vehicle’s body reference frame. When the quadcopter is in a horizontal, untilted position, this command results in purely vertical forces. Specifically, when all propeller speeds are greater than the hovering speed, indicated by the red color, the quadcopter ascends; when they are less than the hovering speed, indicated by the green color, the quadcopter descends. If all propeller speeds are equal to the hovering speed, the quadcopter hovers in place. Fig 2 provides a visual representation of a quadcopter responding to a throttle command, illustrating these dynamics.
- **Roll:** Roll torque can be produced by either reducing the speed of the two left-side propellers (*ω*_3_ and *ω*_4_) while increasing the speed of the right-side propellers (*ω*_1_ and *ω*_2_), or vice versa. This action generates torque around the x-axis of the vehicle. Specifically, when *ω*_1_ and *ω*_2_ are at higher speeds and *ω*_3_ and *ω*_4_ are at lower speeds, the quadcopter rolls around the x-axis, tilts to the left, and moves in the positive y direction. Conversely, when *ω*_1_ and *ω*_2_ are at lower speeds and *ω*_3_ and *ω*_4_ are at higher speeds, the quadcopter rolls around the x-axis, tilts to the right, and moves in the negative y direction. When executing a roll command, it is essential to maintain the overall effective thrust to ensure that only roll acceleration occurs, enabling the vehicle to maneuver along the y-axis of the body reference frame, as depicted in Fig 3.
- **Pitch:** The process of generating pitching movement is similar to that of rolling, with the difference being the axis of moment generation. In pitching, the movement is achieved by either reducing the speed of the two front propellers (*ω*_1_ and *ω*_4_) while increasing the speed of the rear ones (*ω*_2_ and *ω*_3_), or vice versa. This action results in torque being generated along the y-axis of the body reference frame, causing the quadcopter to move in the x direction, as illustrated in Fig 4. Specifically, when *ω*_1_ and *ω*_4_ are at higher speeds and *ω*_2_ and *ω*_3_ are at lower speeds, the quadcopter pitches around the y-axis, tilts to the rear, and moves in the negative x direction. Conversely, when *ω*_1_ and *ω*_4_ are at lower speeds and *ω*_2_ and *ω*_3_ are at higher speeds, the quadcopter pitches around the y-axis, tilts to the front, and moves in the positive x direction. Similar to the roll command, maintaining a constant effective upward thrust ensures that only pitch acceleration is produced.
- **Yaw:** Yaw movement is accomplished by adjusting the rotation of each pair of propellers, with one pair spinning counterclockwise (*ω*_1_ and *ω*_3_) while the other spins clockwise (*ω*_2_ and *ω*_4_). As a result of the imbalance in overall torque, the quadcopter rotates around the z-axis of its body reference frame, as depicted in Fig 5. It is worth noting that a clockwise-rotating propeller generates torque in the same direction as its rotation. This implies that, when viewed from above, a clockwise-rotating propeller induces counterclockwise torque, referred to as positive torque, on the quadcopter. Conversely, a counterclockwise-rotating propeller creates clockwise torque, termed negative torque, when observed from above. Specifically, when *ω*_1_ and *ω*_3_ are at higher speeds and *ω*_2_ and *ω*_4_ are at lower speeds, the quadcopter generates torque in the clockwise direction along the z-axis. Conversely, when *ω*_1_ and *ω*_3_ are at lower speeds and *ω*_2_ and *ω*_4_ are at higher speeds, the quadcopter generates torque in the counterclockwise direction along the z-axis.

### 2.2 Kinematics of quadcopter

To comprehensively describe the behavior of a quadcopter, it is crucial to establish two key reference frames: the Body reference frame (*x*_*B*_, *y*_*B*_, *z*_*B*_) and the Inertial reference frame (*x*_*I*_, *y*_*I*_, *z*_*I*_). The coordinates of the quadcopter can be expressed as a vector *q*, which consists of two components: *η*^*I*^ and *ζ*^*I*^.

The component *η*^*I*^ represents the position coordinates of the quadcopter in the inertial frame, which are *x*, *y*, and *z*. The component *ζ*^*I*^ represents the orientation angles of the quadcopter in the inertial frame, which are the roll (*ϕ*), pitch (*θ*), and yaw (*ψ*) angles.

**Assumption 2.1:** The quadcopter is considered a rigid and inflexible structure, exhibiting axis-symmetrical properties. Furthermore, the center of mass of the quadcopter coincides exactly with the origin of its body frame.

The kinematic equations can be formulated as follows:

$$\begin{array}{c} {\dot{\eta }}^{I}={(}_{B}^{I}R)V \end{array}$$ (1)

where, *V* represents the linear velocity in the B-frame, ${\dot{\eta }}^{I}$ signifies the linear velocity in the I-frame, and *R* denotes the rotation matrix.

BIR=[cos(θ)cos(ψ)sin(ϕ)sin(θ)cos(ψ)-cos(ϕ)sin(ψ)sin(ϕ)sin(ψ)+cos(ϕ)sin(θ)cos(ψ)cos(θ)sin(ψ)cos(ϕ)cos(ψ)+sin(ϕ)sin(θ)sin(ψ)cos(ϕ)sin(θ)sin(ψ)-sin(ϕ)cos(ψ)-sin(θ)sin(ϕ)cos(θ)cos(ϕ)cos(θ)] (2)

The rotational motion of the quadcopter is derived as follows:

$$\begin{array}{c} {\dot{\zeta }}^{I}=T\omega^{B} \end{array}$$ (3)

where, ${\dot{\zeta }}^{I}$ denotes the angular velocity within the I-frame, ***ω***^*B*^ denotes the angular velocity within the B-frame, and T is the transfer matrix (7).

$$\begin{array}{c} T=[\begin{array}{ccc} 1 & \;\text{sin}\;\phi \;\text{tan}\;\theta & \;\text{cos}\;\phi \;\text{tan}\;\theta \\ 0 & \;\text{cos}\;\phi & -\;\text{sin}\;\phi \\ 0 & \;\text{sin}\;\phi /\;\text{cos}\;\theta & \;\text{cos}\;\phi /\;\text{cos}\;\theta \end{array}] \end{array}$$ (4)

### 2.3 Dynamics of quadcopter

The description of the quadcopter dynamics in six degrees of freedom (6-DOF) is provided as follows:

$$\{\begin{array}{c} \ddot{x}=(\;\text{cos}\;\phi \;\text{sin}\;\theta \;\text{cos}\;\psi +\;\text{sin}\;\phi \;\text{sin}\;\psi )\left(\frac{u_{1}}{m}\right)\\ \ddot{y}=(\;\text{cos}\;\phi \;\text{sin}\;\theta \;\text{sin}\;\psi -\;\text{sin}\;\phi \;\text{cos}\;\psi )\left(\frac{u_{1}}{m}\right)\\ \ddot{z}=(\;\text{cos}\;\phi \;\text{cos}\;\theta )\left(\frac{u_{1}}{m}\right)-g\\ \ddot{\phi }=\frac{u_{2}}{I_{xx}}+\frac{\left(I_{yy}-I_{zz}\right)}{I_{xx}}\dot{\theta }\dot{\psi }+\frac{J\dot{\theta }\omega_{r}}{I_{xx}}\\ \ddot{\theta }=\frac{u_{3}}{I_{yy}}+\frac{\left(I_{zz}-I_{xx}\right)}{I_{yy}}\dot{\phi }\dot{\psi }-\frac{J\dot{\phi }\omega_{r}}{I_{yy}}\\ \ddot{\psi }=\frac{u_{4}}{I_{zz}}+\frac{\left(I_{xx}-I_{yy}\right)}{I_{zz}}\dot{\phi }\dot{\theta } \end{array}$$ (5)

where, *ω*_*r*_ = −*ω*_1_ + *ω*_2_ − *ω*_3_ + *ω*_4_ and, The angular velocities can be described as:

$$\{\begin{array}{c} \omega_{1}=\sqrt{\frac{u_{1}}{4k_{t}}+\sqrt{2}\frac{u_{2}}{4k_{t}l}+\sqrt{2}\frac{u_{3}}{4k_{t}l}-\frac{u_{4}}{4k_{\tau }}}\\ \omega_{2}=\sqrt{\frac{u_{1}}{4k_{t}}+\sqrt{2}\frac{u_{2}}{4k_{t}l}-\sqrt{2}\frac{u_{3}}{4k_{t}l}+\frac{u_{4}}{4k_{\tau }}}\\ \omega_{3}=\sqrt{\frac{u_{1}}{4k_{t}}-\sqrt{2}\frac{u_{2}}{4k_{t}l}-\sqrt{2}\frac{u_{3}}{4k_{t}l}-\frac{u_{4}}{4k_{\tau }}}\\ \omega_{4}=\sqrt{\frac{u_{1}}{4k_{t}}-\sqrt{2}\frac{u_{2}}{4k_{t}l}+\sqrt{2}\frac{u_{3}}{4k_{t}l}+\frac{u_{4}}{4k_{\tau }}} \end{array}$$ (6)

where, *k*_*t*_ denotes the propeller thrust coefficient and *k*_*τ*_ denotes the drag coefficient.

### 2.4 State space representation

A state-space representation is a method for characterizing the dynamics of a physical system using state variables and first-order differential equations [55]. Let the state variables be: *x*, $\dot{x}$, *y*, $\dot{y}$, *z*, $\dot{z}$, *ϕ*, $\dot{\phi }$, *θ*, $\dot{\theta }$, *ψ*, and $\dot{\psi }$, denoted as *x*_1_, *x*_2_, *x*_3_, *x*_4_, *x*_5_, *x*_6_, *x*_7_, *x*_8_, *x*_9_, *x*_10_, *x*_11_, and *x*_12_.

The state-space representation for the quadcopter is as follows:

$$\begin{array}{c} \left\{ \begin{array}{cc} {\dot{x}}_{1} & =x_{2}\\ {\dot{x}}_{2} & =\frac{u_{x}}{m}\\ {\dot{x}}_{3} & =x_{4}\\ {\dot{x}}_{4} & =\frac{u_{y}}{m}\\ {\dot{x}}_{5} & =x_{6}\\ {\dot{x}}_{6} & =\frac{u_{z}}{m}\\ {\dot{x}}_{7} & =x_{8}\\ {\dot{x}}_{8} & =b_{1}u_{2}+a_{1}x_{10}x_{12}+a_{2}x_{10}\\ {\dot{x}}_{9} & =x_{10}\\ {\dot{x}}_{10} & =b_{2}u_{3}+a_{3}x_{8}x_{12}-a_{4}x_{8}\\ {\dot{x}}_{11} & =x_{12}\\ {\dot{x}}_{12} & =b_{3}u_{4}+a_{5}x_{8}x_{10} \end{array}\right. \end{array}$$ (7)

$$\begin{array}{c} \text{where},\;a_{1}=\frac{I_{yy}-I_{zz}}{I_{xx}},\;a_{2}=\frac{J\cdot w_{r}}{I_{xx}},\;a_{3}=\frac{I_{zz}-I_{xx}}{I_{yy}}\;a_{4}=\frac{J\cdot w_{r}}{I_{yy}},\;a_{5}=\frac{I_{xx}-I_{yy}}{I_{zz}} \end{array}$$

$$\begin{array}{c} b_{1}=\frac{1}{I_{xx}},\;b_{2}=\frac{1}{I_{yy}},\;b_{3}=\frac{1}{I_{zz}} \end{array}$$

*J* represents the moment of inertia about the propeller axis, while *I*_*xx*_, *I*_*yy*_, and *I*_*zz*_ represent the moments of inertia about the x-axis, y-axis, and z-axis, respectively, when objects are rotated about their respective axes.

**Remark 2.1:** The state-space model for quadcopter systems provides a concise framework for understanding dynamics. By detailing relationships among state variables and incorporating vehicle parameters, this model precisely delineates quadcopter behavior with clarity and accuracy.

## 3 Controller design

**Fig 6:**
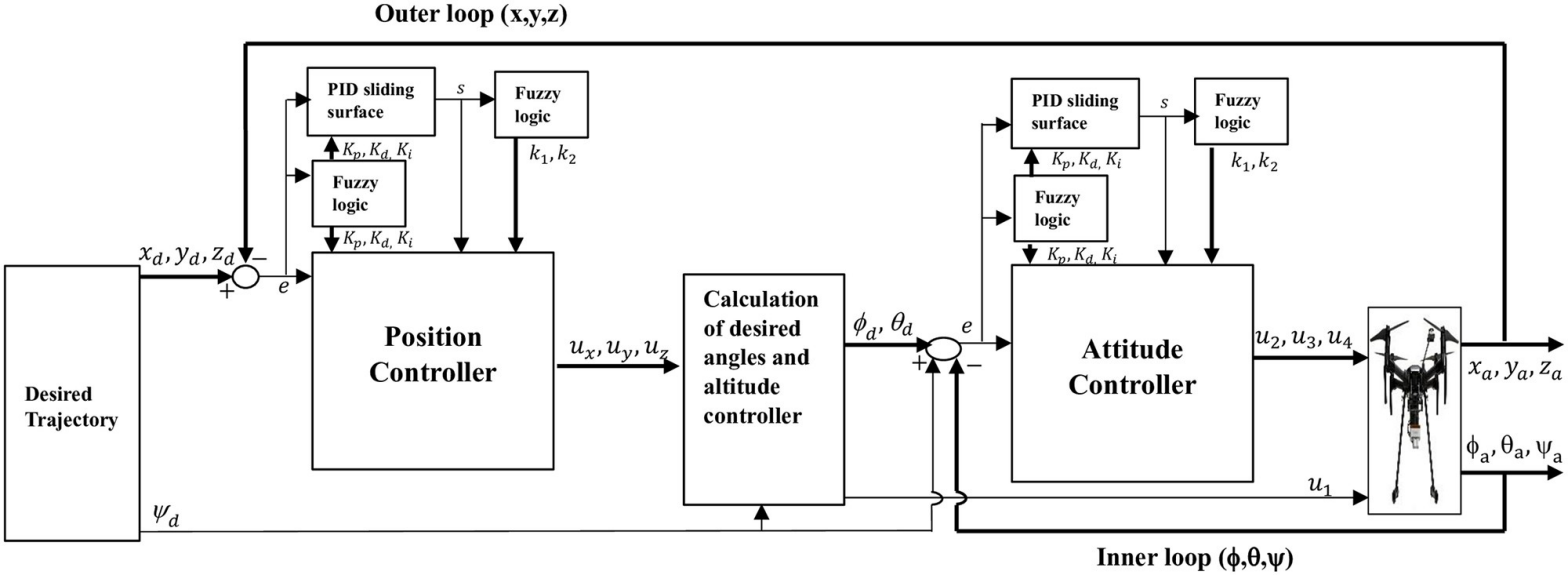
Block diagram of the proposed control strategy.

As depicted in Fig 6, the proposed fuzzy super twisting sliding mode control with fuzzy PID surface is designed to control the trajectory tracking of quadcopter attitude and position.

The process begins with a predefined desired trajectory. The system calculates the error by comparing this trajectory with the actual position and sends this error to a fuzzy logic controller. The fuzzy logic controller uses the error to determine the appropriate values for the control parameters (*K*_*p*_, *K*_*i*_, *K*_*d*_). These parameters are then applied to the sliding surface, which computes additional values. Subsequently, this sliding surface data enters another fuzzy logic controller, where it influences parameters *k*_1_ and *k*_2_. These values, *K*_*p*_, *K*_*i*_, *K*_*d*_, *k*_1_, and *k*_2_, are then integrated into the position controller, which, in turn, generates virtual control inputs (*u*_*x*_, *u*_*y*_, *u*_*z*_). These virtual control inputs are further transformed into desired angles (*ϕ* and *θ*), and attitude controller. These angles serve as inputs to an inner loop controller, where they are subtracted from the actual orientation to produce an error signal. To tune the parameters of the inner loop controller, like the outer loop controller, Fuzzy logic is employed. The resulting parameters are sent to the attitude controller, which outputs *u*_2_, *u*_3_, and *u*_4_ inputs. The control inputs (*u*_1_, *u*_2_, *u*_3_, and *u*_4_) are then applied to the quadcopter model, which delivers the actual position (*x*, *y*, *z*) and orientation (*ϕ*, *θ*, *ψ*) of the quadcopter, as shown in Fig 6.

Since the dynamic equations of the quadcopter in (5) represent an underactuated system, it is controlled by four input variables denoted as *u*_1_, *u*_2_, *u*_3_, and *u*_4_, while exhibiting six degrees of freedom represented as *x*, *y*, *z*, *ϕ*, *θ*, and *ψ*. To achieve independent control, it becomes necessary to transform the underactuated system into a fully actuated one. Assuming no external forces act on the quadcopter, only the thrust force and gravitational force are considered.

Consider the virtual control inputs in the x, y, and z directions, denoted as *u*_*x*_, *u*_*y*_, and *u*_*z*_.

$$\begin{array}{c} {\left[\begin{array}{ccc} u_{x} & u_{y} & u_{z} \end{array}\right]}^{T}{=}_{B}^{I}R{\left[\begin{array}{ccc} 0 & 0 & u_{1} \end{array}\right]}^{T}-{\left[\begin{array}{ccc} 0 & 0 & mg \end{array}\right]}^{T} \end{array}$$ (8)

where

$$\begin{array}{c} \left[\begin{array}{c} u_{x}\\ u_{y}\\ u_{z} \end{array}]=[\begin{array}{c} \ddot{x}\\ \ddot{y}\\ \ddot{z} \end{array}\right] \end{array}$$

Eq (8) can be expressed in the following manner:

$$\begin{array}{c} \left\{ \begin{array}{cc} u_{x} & =\left(\;\text{sin}\;\left(\phi \right)\;\text{sin}\;\left(\psi \right)+\;\text{cos}\;\left(\phi \right)\;\text{sin}\;\left(\theta \right)\;\text{cos}\;\left(\psi \right)\right)u_{1}\\ u_{y} & =\left(-\;\text{sin}\;\left(\phi \right)\;\text{cos}\;\left(\psi \right)+\;\text{cos}\;\left(\phi \right)\;\text{sin}\;\left(\theta \right)\;\text{sin}\;\left(\psi \right)\right)u_{1}\\ u_{z} & =\left(\;\text{cos}\;\left(\phi \right)\;\text{cos}\;\left(\theta \right)\right)u_{1}-mg \end{array}\right. \end{array}$$ (9)

Utilizing virtual controls, the quadcopter’s positions along the *x*, *y*, and *z* axes are managed, incorporating (*u*_1_, *ϕ*_*d*_, and *θ*_*d*_). The outer loop controller acquires the desired position values (*x*_*d*_, *y*_*d*_, *z*_*d*_) and the present trajectory (*x*, *y*, *z*) to generate the virtual controls (*u*_*x*_, *u*_*y*_, *u*_*z*_). Subsequently, the lift force (*u*_1_) is generated, along with the desired roll (*ϕ*_*d*_) and pitch (*θ*_*d*_) angles.

**Assumption 3.1:** It is established that the desired trajectory, denoted as $\eta_{d}^{I}={\left[x_{d},y_{d},z_{d}\right]}^{T}$, along with the desired yaw angle *ψ*_*d*_, are predetermined and accessible. Moreover, both the first and second derivatives are bounded.

**Remark 3.1:** Assumption 3.1 constitutes a justified and moderate supposition, as, in the context of trajectory tracking for the quadcopter, both the desired trajectory and yaw angle invariably vary within a specific range.

### 3.1 SMC with PID surface design

**Lemma 3.1** [56]: For a given control system described by the nonlinear dynamics $\dot{x}=f\left(x\right)+g\left(x\right)u$, where *x* represents the state vector, *u* the control input, and *f*(*x*) and *g*(*x*) known functions, consider a sliding surface defined as *s*(*x*) = 0. If a sliding mode controller *u* is designed such that the sliding condition $s\dot{s}\leq -\phi \left(s\right)$ is satisfied, where *ϕ*(*s*) is a positive definite function, then the state trajectory of the system will converge to the sliding surface *s* = 0 in finite time, and the motion on the sliding surface is stable. This ensures robustness against matched disturbances and uncertainties.

Introduce the position and attitude subsystem tracking errors in the following way:

$$\left\{\begin{array}{c} e_{1}=x_{1}-x_{d}\\ e_{3}=x_{3}-y_{d}\\ e_{5}=x_{5}-z_{d}\\ e_{7}=x_{7}-\phi_{d}\\ e_{9}=x_{9}-\theta_{d}\\ e_{11}=x_{11}-\psi_{d} \end{array}\right.$$ (10)

The PID sliding surfaces are chosen as:

$$\left\{\begin{array}{c} s_{1}=K_{px}e_{1}+K_{ix}\int e_{1}\;dt+K_{dx}{\dot{e}}_{1}\\ s_{3}=K_{py}e_{3}+K_{iy}\int e_{3}\;dt+K_{dy}{\dot{e}}_{3}\\ s_{5}=K_{pz}e_{5}+K_{iz}\int e_{5}\;dt+K_{dz}{\dot{e}}_{5}\\ s_{7}=K_{p\phi }e_{7}+K_{i\phi }\int e_{7}\;dt+K_{d\phi }{\dot{e}}_{7}\\ s_{9}=K_{p\theta }e_{9}+K_{i\theta }\int e_{9}\;dt+K_{d\theta }{\dot{e}}_{9}\\ s_{11}=K_{p\psi }e_{11}+K_{i\psi }\int e_{11}\;dt+K_{d\psi }{\dot{e}}_{11} \end{array}\right.$$ (11)

**Lemma 3.2** [57]: Once the condition $s=\dot{s}=0$ for the sliding manifold is met, where $\dot{s}={\left({\dot{s}}_{11},\ldots ,{\dot{s}}_{1m},\ldots ,{\dot{s}}_{n1},\ldots ,{\dot{s}}_{nm}\right)}^{T}$, it is established that the absolute state error converges to zero within a finite time.

The derivative of the PID sliding surfaces with respect to time is expressed as:

$$\left\{\begin{array}{c} {\dot{s}}_{1}=K_{px}{\dot{e}}_{1}+K_{ix}e_{1}+K_{dx}{\ddot{e}}_{1}\\ {\dot{s}}_{3}=K_{py}{\dot{e}}_{3}+K_{iy}e_{3}+K_{dy}{\ddot{e}}_{3}\\ {\dot{s}}_{5}=K_{pz}{\dot{e}}_{5}+K_{iz}e_{5}+K_{dz}{\ddot{e}}_{5}\\ {\dot{s}}_{7}=K_{p\phi }{\dot{e}}_{7}+K_{i\phi }e_{7}+K_{d\phi }{\ddot{e}}_{7}\\ {\dot{s}}_{9}=K_{p\theta }{\dot{e}}_{9}+K_{i\theta }e_{9}+K_{d\theta }{\ddot{e}}_{9}\\ {\dot{s}}_{11}=K_{p\psi }{\dot{e}}_{11}+K_{i\psi }e_{11}+K_{d\psi }{\ddot{e}}_{11} \end{array}\right.$$ (12)

The switching control laws for both the translational and rotational subsystems are based on the constant rate reaching law:

$$\left\{\begin{array}{c} {\dot{s}}_{1}=-Q_{x}\text{sign}\left(s_{1}\right)\\ {\dot{s}}_{3}=-Q_{y}\text{sign}\left(s_{3}\right)\\ {\dot{s}}_{5}=-Q_{z}\text{sign}\left(s_{5}\right)\\ {\dot{s}}_{7}=-Q_{\phi }sign\left(s_{7}\right)\\ {\dot{s}}_{9}=-Q_{\theta }\text{sign}\left(s_{9}\right)\\ {\dot{s}}_{11}=-Q_{\psi }\text{sign}\left(s_{11}\right) \end{array}\right.$$ (13)

From Eqs (12) and (13) the entire control law for position and attitude is given by:

$$\begin{array}{c} \left\{ \begin{array}{cc} & u_{x}=\frac{m}{K_{d_{x}}}(-Q_{x}\text{sign}\left(s_{1}\right)+K_{d_{x}}{\ddot{x}}_{d}-K_{p_{x}}{\dot{e}}_{1}-K_{i_{x}}e_{1})\\ & u_{y}=\frac{m}{K_{d_{y}}}(-Q_{y}\text{sign}\left(s_{3}\right)+K_{d_{y}}{\ddot{y}}_{d}-K_{p_{y}}{\dot{e}}_{3}-K_{i_{y}}e_{3})\\ & u_{z}=\frac{m}{K_{d_{z}}}(-Q_{z}\text{sign}\left(s_{5}\right)+K_{d_{z}}{\ddot{z}}_{d}-K_{p_{z}}{\dot{e}}_{5}-K_{i_{z}}e_{5})\\ & u_{2}=\frac{1}{K_{d_{\phi }}b_{1}}(-Q_{\phi }\text{sign}\left(s_{7}\right)+K_{d_{\phi }}{\ddot{\phi }}_{d}-K_{p_{\phi }}{\dot{e}}_{7}-K_{i_{\phi }}e_{7}\\ & \;\;-K_{d_{\phi }}a_{1}x_{10}x_{12}-a_{2}x_{10}K_{d_{\phi }})\\ & u_{3}=\frac{1}{K_{d_{\theta }}b_{2}}(-Q_{\theta }\text{sign}\left(s_{9}\right)+K_{d_{\theta }}{\ddot{\theta }}_{d}-K_{p_{\theta }}{\dot{e}}_{9}-K_{i_{\theta }}e_{9}\\ & \;\;-K_{d_{\theta }}a_{3}x_{8}x_{12}+a_{4}x_{8}K_{d_{\phi }})\\ & u_{4}=\frac{1}{K_{d_{\psi }}b_{3}}(-Q_{\psi }\text{sign}\left(s_{11}\right)+K_{d_{\psi }}{\ddot{\psi }}_{d}-K_{p_{\psi }}{\dot{e}}_{11}-K_{i_{\psi }}e_{11}\\ & \;\;-K_{d_{\psi }}a_{5}x_{8}x_{10}) \end{array}\right. \end{array}$$ (14)

where *Q*_*j*_ for *j* = (*x*, *y*, *z*, *ϕ*, *θ*, *ψ*) is a positive parameter.

#### 3.1.1 Stability analysis

**Theorem 3.1:** Consider an autonomous system defined by the differential equation $\dot{x}=f\left(x\right)$, where *x* = 0 represents an equilibrium point, and the function $f:R^{n}\to R^{n}$ is locally Lipschitz in a neighborhood around the origin, ensuring uniqueness and existence of solutions, with *f*(0) = 0. Let $V:R^{n}\to R$ be a continuously differentiable function, known as the Lyapunov function, satisfying the following conditions within a neighborhood *D* surrounding the origin:

1. *V*(*x*) is positive definite, i.e., *V*(*x*)>0 for all *x* ≠ 0 within *D*, and *V*(0) = 0.
2. The time derivative of *V*(*x*), denoted by $\dot{V}\left(x\right)=\nabla V\left(x\right)\cdot f\left(x\right)$, is negative semi-definite, i.e., $\dot{V}\left(x\right)\leq 0$ for all *x* in *D*.

Under these conditions, the equilibrium point *x* = 0 is deemed stable. Furthermore, if $\dot{V}\left(x\right)$ is negative definite, meaning $\dot{V}\left(x\right)<0$ for all *x* ≠ 0 in *D*, then the equilibrium point is classified as asymptotically stable.

**Remark 1:** The proof provided in S1 Appendix demonstrates that the designed sliding mode controller with PID surface ensures that the system is asymptotically stable. The Lyapunov candidate function used in the proof confirms that the system’s state trajectory converges to the equilibrium point *x* = 0, and the state errors diminish over time.

### 3.2 STSMC with PID surface design

**Lemma 3.3** [35]: Consider a dynamical system controlled by a super-twisting sliding mode controller. If the control law is designed such that the sliding variable *s* and its time derivative $\dot{s}$ satisfy the super-twisting algorithm conditions, then the state trajectory of the system will reach the sliding surface *s* = 0 in finite time, ensuring robustness against uncertainties and external disturbances. Moreover, the reaching condition $s\dot{s}<0$ is fulfilled, guaranteeing the system’s stability and reducing the chattering effect inherent to conventional sliding mode control.

The super twisting sliding mode controller is the summation of the equivalent and continuous controller:

$$\begin{array}{c} u_{\text{STSMC}}=u_{\text{eq}}+u_{\text{ST}} \end{array}$$ (15)

The equivalent controller *u*_eq_ remains consistent with the SMC design. Based on the super-twisting algorithm, the closed-loop sliding dynamics are set as:

$$\left\{\begin{array}{c} {\dot{s}}_{1}=-k_{1}|s_{1}{|}^{1/2}\text{sign}(s_{1})-k_{2}\int \text{sign}(s_{1})dt\\ {\dot{s}}_{3}=-k_{1}|s_{3}{|}^{1/2}\text{sign}(s_{3})-k_{2}\int \text{sign}(s_{3})dt\\ {\dot{s}}_{5}=-k_{1}|s_{5}{|}^{1/2}\text{sign}(s_{5})-k_{2}\int \text{sign}(s_{5})dt\\ {\dot{s}}_{7}=-k_{1}|s_{7}{|}^{1/2}\text{sign}(s_{7})-k_{2}\int \text{sign}(s_{7})dt\\ {\dot{s}}_{9}=-k_{1}|s_{9}{|}^{1/2}\text{sign}(s_{9})-k_{2}\int \text{sign}(s_{9})dt\\ {\dot{s}}_{11}=-k_{1}|s_{11}{|}^{1/2}\text{sign}(s_{11})-k_{2}\int \text{sign}(s_{11})dt \end{array}\right.$$ (16)

From Eqs (12) and (16) the position and attitude super twisting SMC of the quadcopter is summarized as:

$$\begin{array}{c} \left\{ \begin{array}{cc} u_{x} & =\frac{m}{K_{dx}}(-k_{1}\sqrt{|s_{1}|}\text{sign}\left(s_{1}\right)-k_{2}\int \text{sign}\left(s_{1}\right)\;dt+K_{dx}{\ddot{x}}_{d}-K_{px}{\dot{e}}_{1}-K_{ix}e_{1})\\ u_{y} & =\frac{m}{K_{dy}}(-k_{1}\sqrt{|s_{3}|}\text{sign}\left(s_{3}\right)-k_{2}\int \text{sign}\left(s_{3}\right)\;dt+K_{dy}{\ddot{y}}_{d}-K_{py}{\dot{e}}_{3}-K_{iy}e_{3})\\ u_{z} & =\frac{m}{K_{dz}}(-k_{1}\sqrt{|s_{5}|}\text{sign}\left(s_{5}\right)-k_{2}\int \text{sign}\left(s_{5}\right)\;dt+K_{dz}{\ddot{z}}_{d}-K_{pz}{\dot{e}}_{5}-K_{iz}e_{5})\\ u_{2} & =\frac{1}{K_{d\phi }b_{1}}(-k_{1}\sqrt{|s_{7}|}\text{sign}\left(s_{7}\right)-k_{2}\int \text{sign}\left(s_{7}\right)\;dt+K_{d\phi }{\ddot{\phi }}_{d}-K_{p\phi }{\dot{e}}_{7}-K_{i\phi }e_{7}\\ & \;\;-K_{d\phi }a_{1}x_{10}x_{12}-a_{2}x_{10}K_{d\phi })\\ u_{3} & =\frac{1}{K_{d\theta }b_{2}}(-k_{1}\sqrt{|s_{9}|}\text{sign}\left(s_{9}\right)-k_{2}\int \text{sign}\left(s_{9}\right)\;dt+K_{d\theta }{\ddot{\theta }}_{d}-K_{p\theta }{\dot{e}}_{9}-K_{i\theta }e_{9}\\ & \;\;-K_{d\theta }a_{3}x_{8}x_{12}+a_{4}x_{8}K_{d\phi })\\ u_{4} & =\frac{1}{K_{d\psi }b_{3}}(-k_{1}\sqrt{|s_{11}|}\text{sign}\left(s_{11}\right)-k_{2}\int \text{sign}\left(s_{11}\right)\;dt+K_{d\psi }{\ddot{\psi }}_{d}-K_{p\psi }{\dot{e}}_{11}\\ & \;\;-K_{i\psi }e_{11}-K_{d\psi }a_{5}x_{8}x_{10}) \end{array}\right. \end{array}$$ (17)

where, *k*_1_ and *k*_2_ are positive constants.

#### 3.2.1 Stability analysis

The stability of the super-twisting sliding mode controller can be analyzed using the Lyapunov candidate function $V\left(s_{1}\right)=\frac{1}{2}s_{1}^{2}$. The time derivative $\dot{V}\left(s_{1}\right)$ is negative definite, indicating that the system is asymptotically stable. This ensures that the state trajectory will reach the sliding surface *s* = 0 in finite time, demonstrating robustness against uncertainties and disturbances.

**Remark 2:** The proof provided in S2 Appendix shows that the super-twisting sliding mode controller ensures the system’s robustness and stability. The Lyapunov candidate function used in this analysis confirms that the system’s state trajectory will reach the sliding surface in finite time and the state errors will diminish, making the system asymptotically stable.

### 3.3 Parameters tuning

Since STSMC has high control effort and complexity in parameter tuning problem then, a careful selection of the control parameters is crucial to strike a balance between achieving fast convergence and minimizing overshoot. Using fixed PID sliding surface parameters in a sliding mode controller has low performance and sensitivity to parameter selection problem. Selecting appropriate fixed parameters is challenging. This sensitivity to parameter selection makes it difficult to achieve the desired control performance consistently. Tuning parameters using fuzzy logic enables the automatic adjustment of the controller’s parameters based on the system’s dynamics and the perturbations encountered during operation. To address such problem of tuning parameter, Fuzzy logic based gain tuning for PID surfaces and super twisting parameters has been used in this paper.

#### 3.3.1 Fuzzy logic-based gain tuning for PID sliding surfaces

Using large PID sliding surface parameters in STSMC for controlling a quadcopter when the error is small introduces overshoot and increased control effort. Using small PID sliding surface parameters in STSMC for controlling a quadcopter when the error is large brings slower convergence problem.

A large value *K*_*p*_, *K*_*i*_, and *K*_*d*_ can be selected when |*e*| is large, to make the control input high and able to converge fast. A small value of *K*_*p*_, *K*_*i*_, and *K*_*d*_ can be selected when |*e*| is small, to decrease overshoot and control effort. Therefore, if the parameters can be tuned based on the above argument, a more acceptable performance (fast convergence, small control effort, and eliminate overshoot) can be achieved. Thus, to have a value of *K*_*p*_, *K*_*i*_, and *K*_*d*_ which can maintain a balance between the two facts, a fuzzy controller rule base is used for tuning *K*_*p*_, *K*_*i*_, and *K*_*d*_ based on |*e*| value. A one-input three-output fuzzy system is designed for this application. The rule base used is given in Table 1. Input |*e*| has four membership functions, and output *K*_*p*_, *K*_*i*_, and *K*_*d*_ also have four membership functions for each.

**Table 1:** Fuzzy rules for error and PID sliding surface parameters.

|*e*|	ZR	PS	PM	PB
*K*_*p*_	ZR	PS	PM	PB
*K*_*i*_	ZR	PS	PM	PB
*K*_*d*_	ZR	PS	PM	PB

Four fuzzy sets named ZR, PS, PM, and PB respectively for Zero, Positive Small, Positive Medium, and Positive Big are chosen to fuzzify the error. Similarly, the parameters *K*_*p*_, *K*_*i*_, and *K*_*d*_ are fuzzified into four fuzzy sets named ZR, PS, PM, and PB respectively for Zero, Positive Small, Positive Medium, and Positive Big. The rules used to map the input and output fuzzy sets are as follows:

**Rule1:** if |*e*| is ZR, then *K*_*p*_, *K*_*i*_, and *K*_*d*_ is ZR.
**Rule2:** if |*e*| is PS, then *K*_*p*_, *K*_*i*_, and *K*_*d*_ is PS.
**Rule3:** if |*e*| is PM, then *K*_*p*_, *K*_*i*_, and *K*_*d*_ is PM.
**Rule4:** if |*e*| is PB, then *K*_*p*_, *K*_*i*_, and *K*_*d*_ is PB.

#### 3.3.2 Fuzzy logic-based gain tuning for *k*_1_ and *k*_2_

Using a large super twisting algorithm parameter in STSMC for controlling a quadcopter when the sliding surface is small introduces high control effort and reduced precision. Using small super twisting algorithm parameters in STSMC for controlling a quadcopter when the sliding surface is large introduces slow convergence and reduced controlability problem.

A large value *k*_1_ and *k*_2_ can be selected when |*s*| is large, to increase control authority and able to converge fast. A small value of *k*_1_ and *k*_2_ can be selected when |*s*| is small, to decrease control effort and increase precision. Therefore, if the parameters can be tuned based on the above argument, a more acceptable performance (fast convergence, small control effort, best precision, and increased control authority) can be achieved. Thus, to have a value of *k*_1_ and *k*_2_ which can maintain a balance between the two facts, a fuzzy controller rule base is used for tuning *k*_1_ and *k*_2_ based on |*s*| value. A one-input two-output fuzzy system is designed for this application. Input taken is |*s*| while the output is *k*_1_ and *k*_2_. The rule base used is given in Table 2. Input |*s*| has five membership functions, and output *k*_1_ and *k*_2_ also have five membership functions for each.

**Table 2:** Fuzzy rules for sliding surface and super twisting algorithm parameters.

|*s*|	VS	S	M	L	VL
*k*_1_	VS	S	M	L	VL
*k*_2_	VS	S	M	L	VL

Five fuzzy sets named VS, S, M, L, and VL respectively for Very Small, Small, Medium, Large, and Very Large are chosen to fuzzify the error. Similarly, the parameters *k*_1_ and *k*_2_ are fuzzified into five fuzzy sets named VS, S, M, L, and VL respectively for Very Small, Small, Medium, Large, and Very Large. The rules used to map the input and output fuzzy sets are as follows:

**Rule 1:** if |*s*| is VS, then *k*_1_ and *k*_2_ are VS.
**Rule 2:** if |*s*| is S, then *k*_1_ and *k*_2_ are S.
**Rule 3:** if |*s*| is M, then *k*_1_ and *k*_2_ are M.
**Rule 4:** if |*s*| is L, then *k*_1_ and *k*_2_ are L.
**Rule 5:** if |*s*| is VL, then *k*_1_ and *k*_2_ are VL.

The membership function used here is triangular function.

## 4 Simulation model

The simulation of the quadcopter model was conducted within the Matlab/Simulink environment. The parameters of the quadcopter utilized in this simulation have been adopted from [58] and are detailed in Table 3.

**Table 3:** Parameters of the quadcopter.

Parameter	Symbol	Value (Unit)
Quadcopter mass	*m*	0.650 kg
Inertia constants	*I*_*xx*_ = *I*_*yy*_	7.5 × 10^−3^ kg·m²
	*I*_*zz*_	1.3 × 10^−2^
Thrust coefficient	*k*_*T*_	3.13 × 10^−5^ N·s²
Drag coefficient	*k*_*τ*_	7.5 × 10^−7^ N·m·s²
Inertia about propeller axis	*J*	6 × 10^−5^ kg·m²
Arm length	*l*	0.23 m

## 5. Simulation results and discussion

Graphical representations of simulation results, created using SIMULINK, provide insights into the performance of the proposed controller. This controller integrates the adaptability of fuzzy logic with the robustness of STSMC. Testing includes trajectory tracking of the spiral infinity trajectory, square wave trajectory, and Ramp Helix trajectory, as well as disturbance rejection capacity. Furthermore, a comparison is made with the Helical trajectory.

### 5.1 Spiral infinity trajectory

In the context of space navigation, a spiral infinity trajectory was utilized as the tracking reference, with sinusoidal input trajectories applied to both the x and y axes, and ramp input to z axes: *x*_*d*_ = 1 − cos(*t*), *y*_*d*_ = 0.5sin(2*t*), and *z*_*d*_ = *t*. The gains are automatically tuned using fuzzy logic.

**Fig 7:**
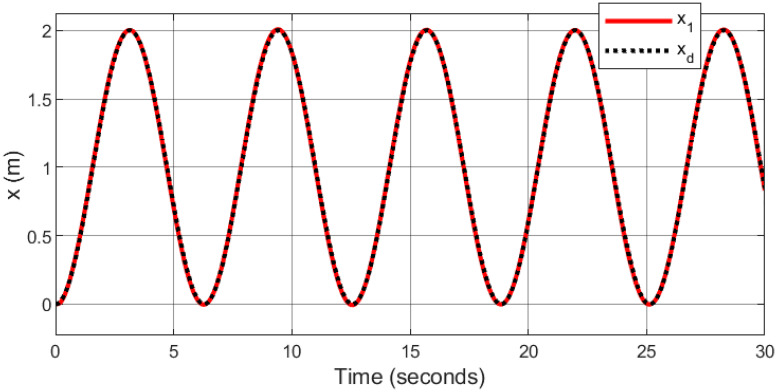
Trajectory tracking for *x*-axis in spiral infinity path.

**Fig 8:**
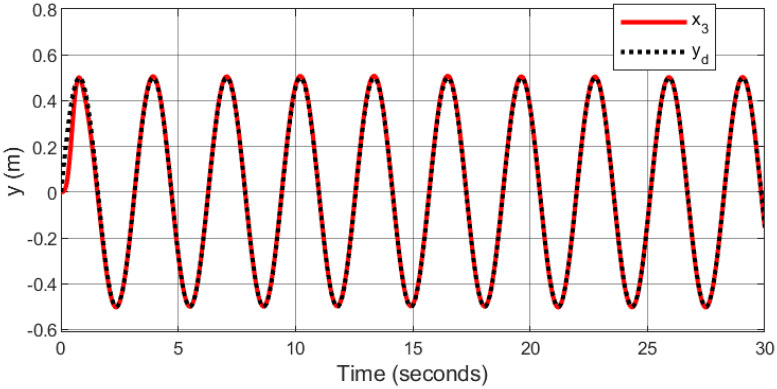
Trajectory tracking for *y*-axis in spiral infinity path.

**Fig 9:**
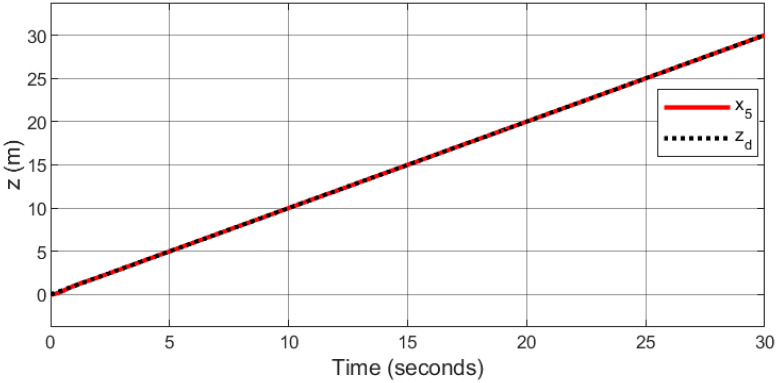
Trajectory tracking for *z*-axis in spiral infinity path.

**Fig 10:**
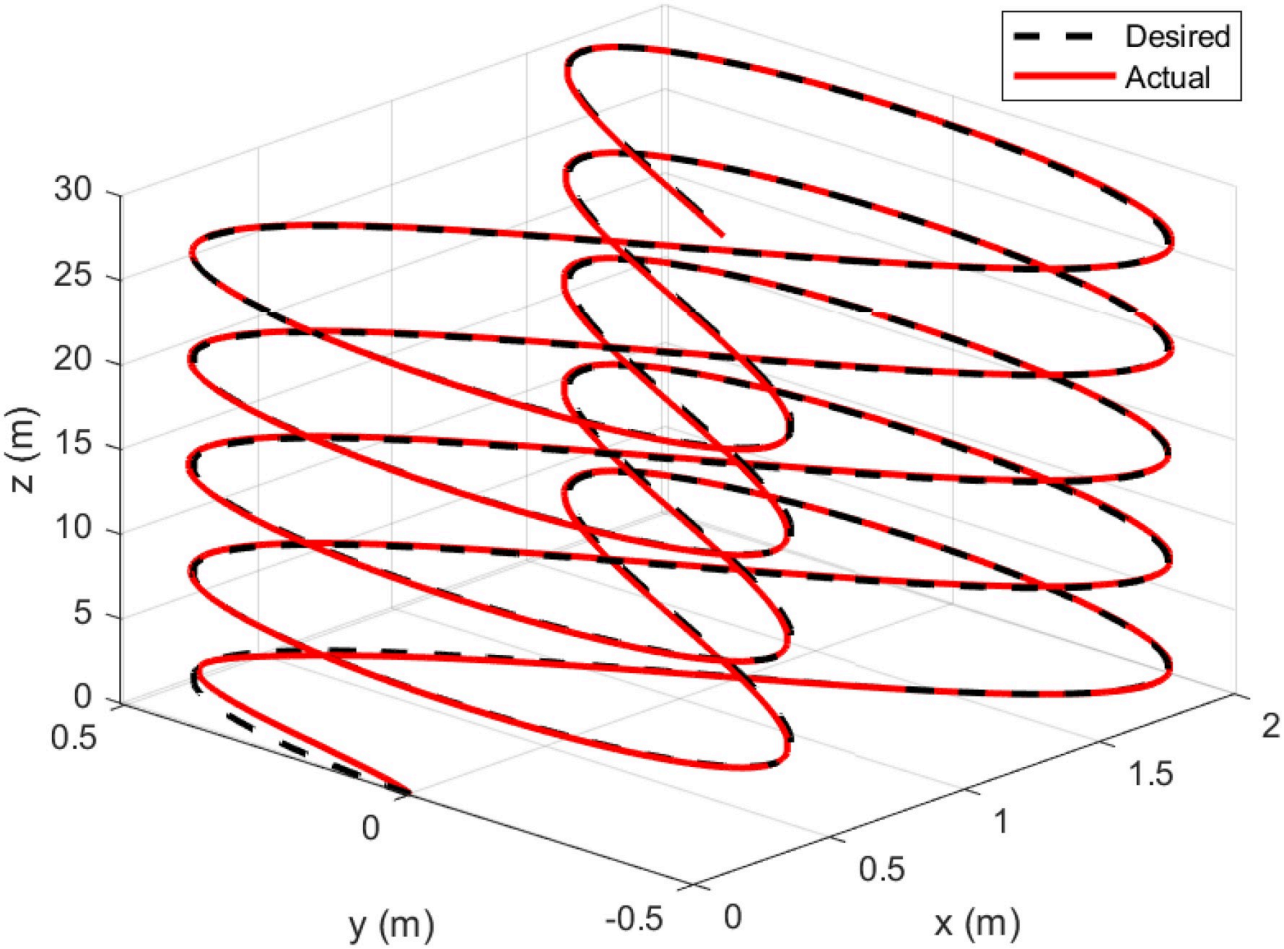
3D trajectory tracking of a spiral infinity path.

Figs 7–10 illustrate the trajectory tracking responses for the *x*, *y*, *z* axes, and the 3D trajectory, respectively. These responses demonstrate that the proposed controller ensures accurate reference tracking, rapid convergence, and maintains system stability.

### 5.2 Square wave trajectory

A square wave trajectory, specifically designed for aerial photography using the quadcopter, was set at an altitude of 5 meters and a speed of 0.5 meters per second. It also featured a 0.1% overlap, thereby ensuring that each image includes a portion of the area documented in a preceding photo.

**Fig 11:**
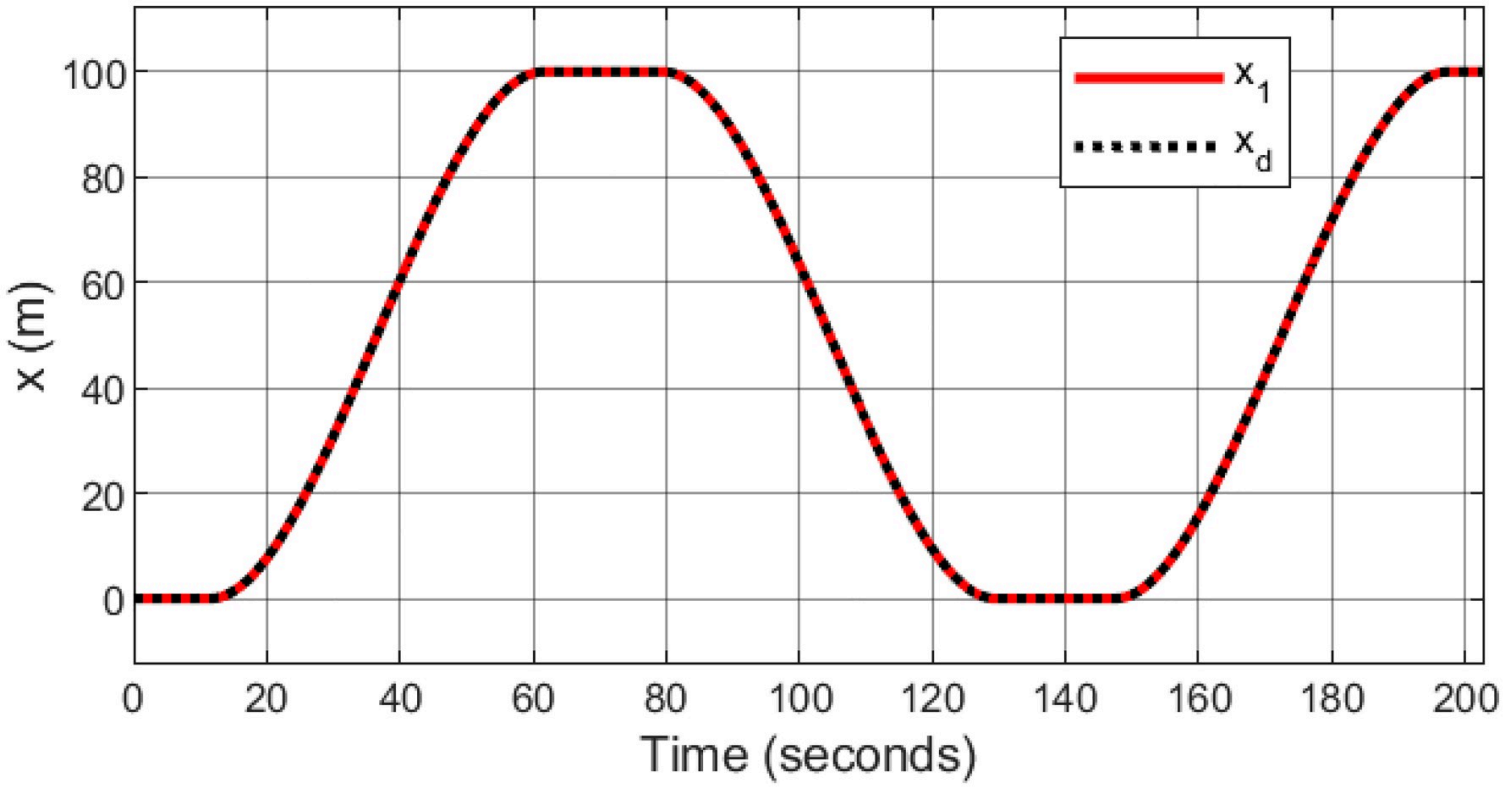
Trajectory tracking for *x*-axis in a square wave path.

**Fig 12:**
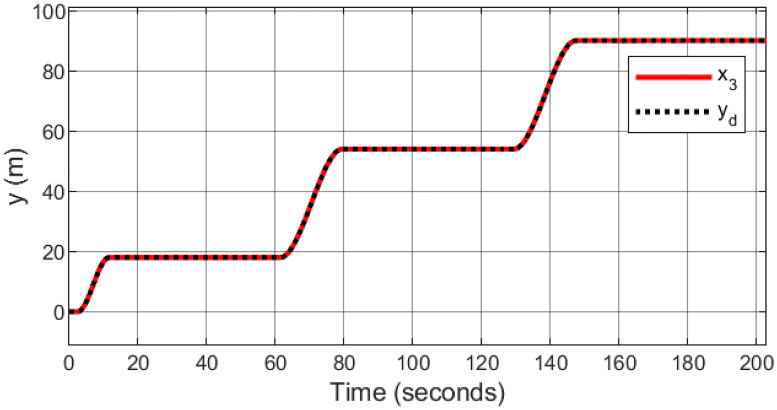
Trajectory tracking for *y*-axis in a square wave path.

**Fig 13:**
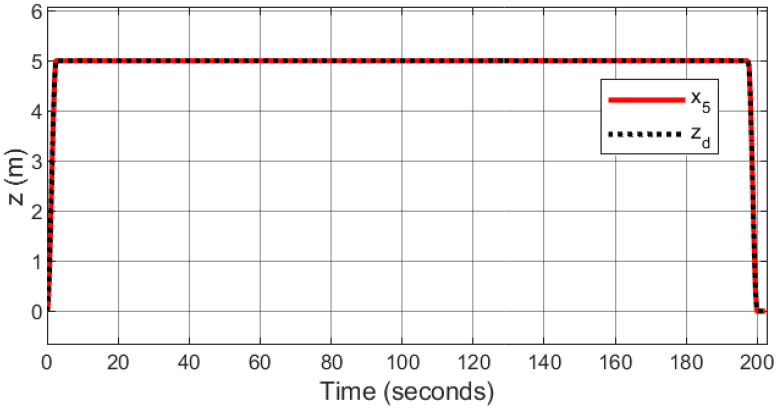
Trajectory tracking for *z*-axis in a square wave path.

**Fig 14:**
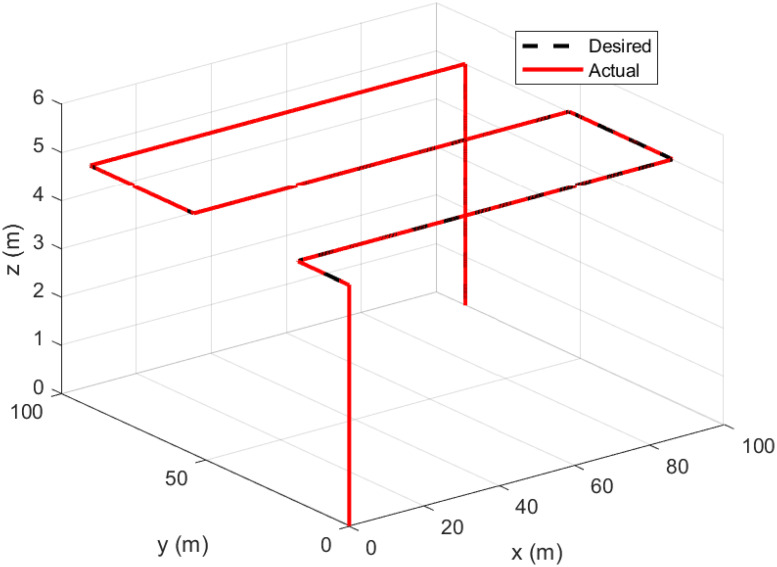
3D trajectory tracking of a square wave path.

Figs 11–14 illustrates the resulting trajectory tracking response to square wave. The responses indicate that the proposed controller effectively tracks the reference and converges fast while maintaining stability.

### 5.3 Ramp helix trajectory

A helical trajectory is generated through the specified desired trajectories: *x*_*d*_ = 1 − cos(*t*), *y*_*d*_ = 1 − cos(*t*), *z*_*d*_ = *t*, and *ψ*_*d*_ = sin(*t*).

**Fig 15:**
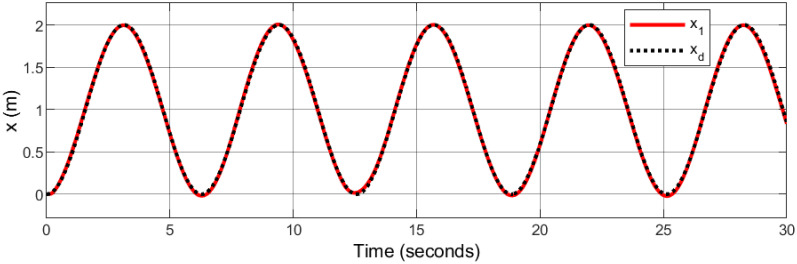
Trajectory tracking for *x*-axis in a ramp helix path.

**Fig 16:**
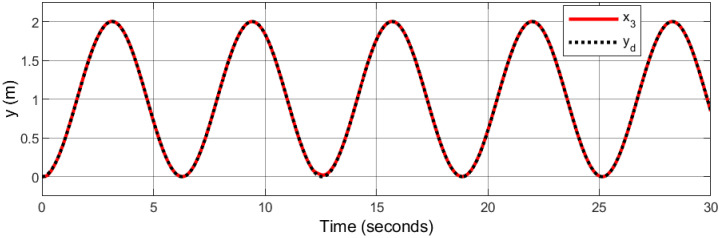
Trajectory tracking for *y*-axis in a ramp helix path.

**Fig 17:**
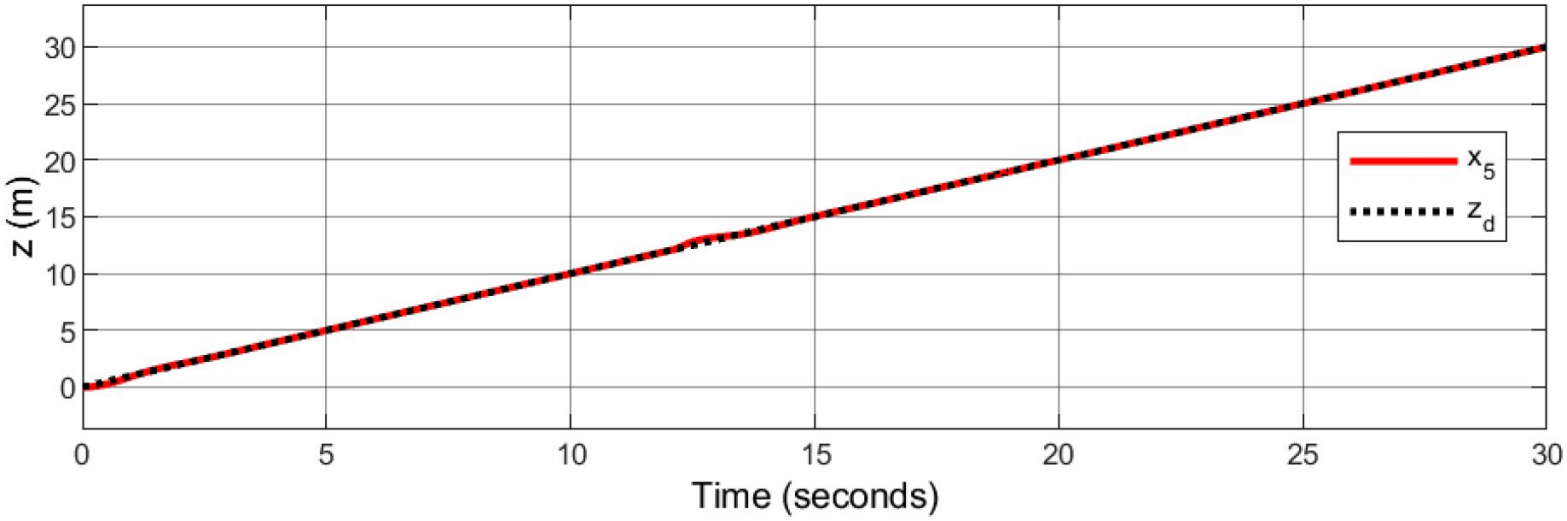
Trajectory tracking for *z*-axis in a ramp helix path.

**Fig 18:**
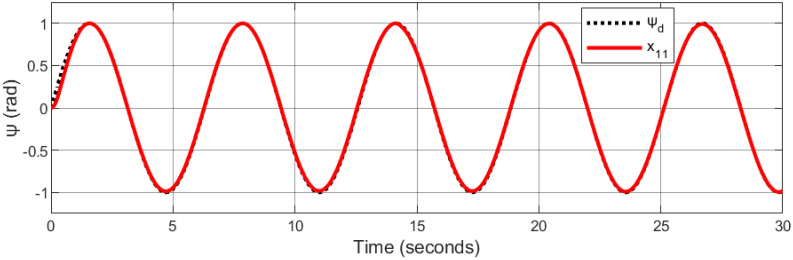
Trajectory tracking for psi in a ramp helix path.

**Fig 19:**
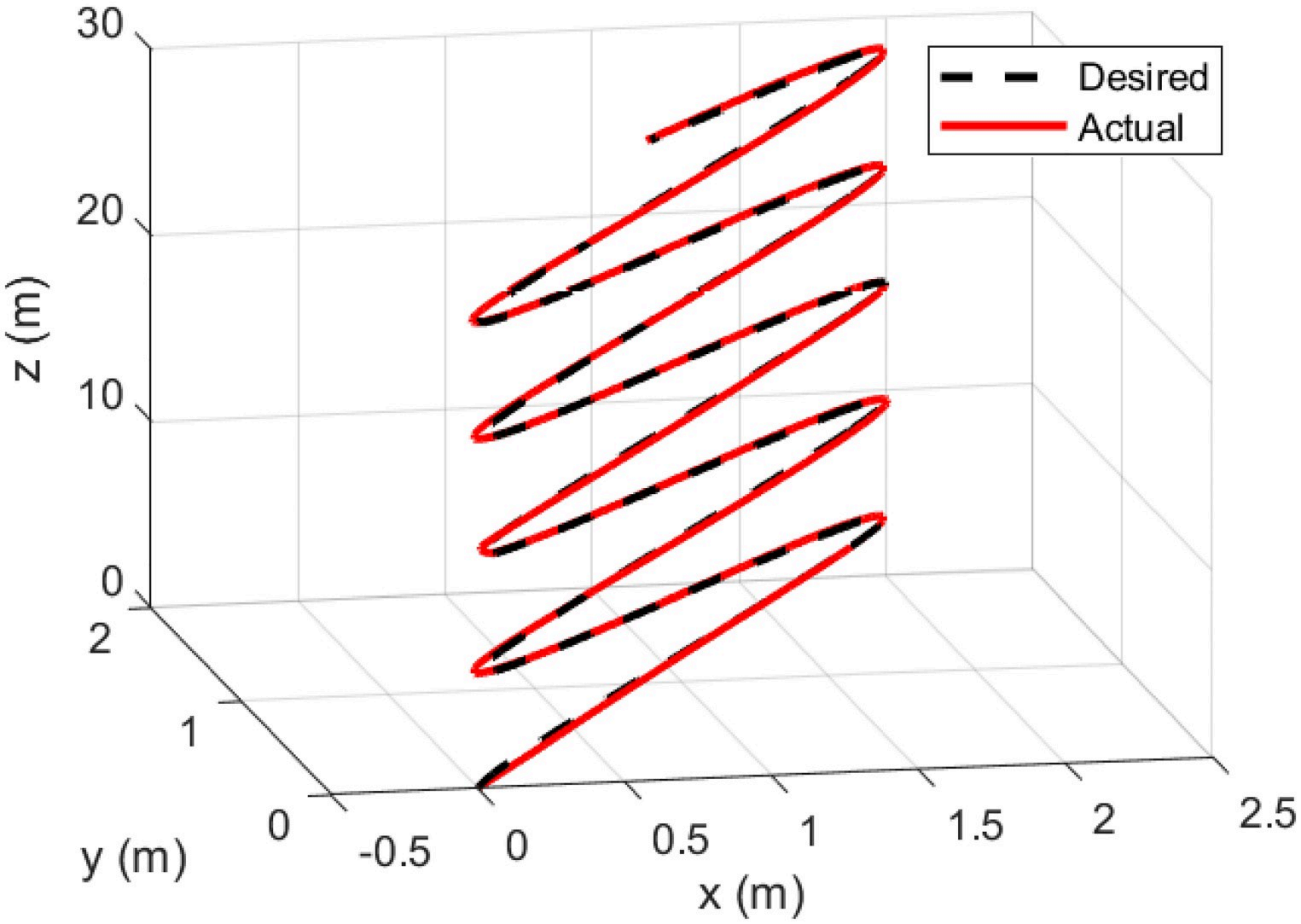
3D trajectory tracking of a ramp helix path.

Figs 15–19 delineate the trajectory tracking response of the ramp helix. The acquired results elucidate the efficacy of the proposed controller in achieving precise reference tracking and rapid convergence, all while upholding system stability. Moreover, they demonstrate successful tracking of the designated yaw trajectory.

### 5.4 Disturbance rejection capacity

In order to rigorously assess the efficiency and robustness of the proposed controller in trajectory tracking, bounded disturbances were applied. The external disturbance is represented by a wind gust with a speed of 5.1444 m/s, resulting in a force of 1.531 N applied along *u*_1_ and a torque of 0.097 Nm applied along *u*_2_ and *u*_3_ from 23 to 26 seconds. Additionally, to examine the effect of wind gusts at different intervals, a wind gust with a speed of 6.687 m/s, resulting in a force of 2.594 N, is applied along *u*_1_ from 8 to 11 seconds. Furthermore, a wind gust with a speed of 4.63 m/s, resulting in a torque of 0.079 Nm, is applied along *u*_2_ from 13 to 16 seconds and along *u*_3_ from 18 to 21 seconds.

**Fig 20:**
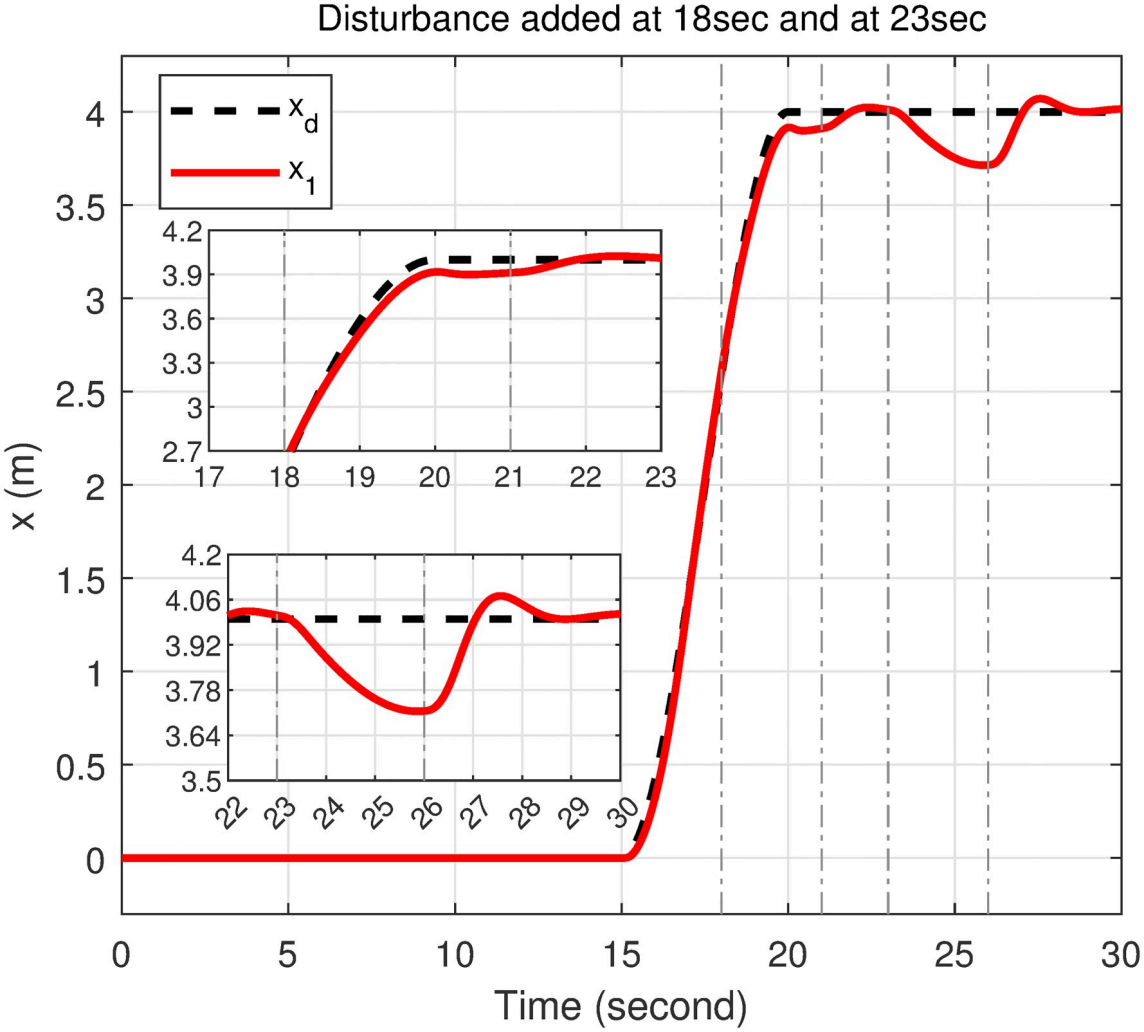
Disturbance rejection capacity of the proposed controller for the *x*-axis.

**Fig 21:**
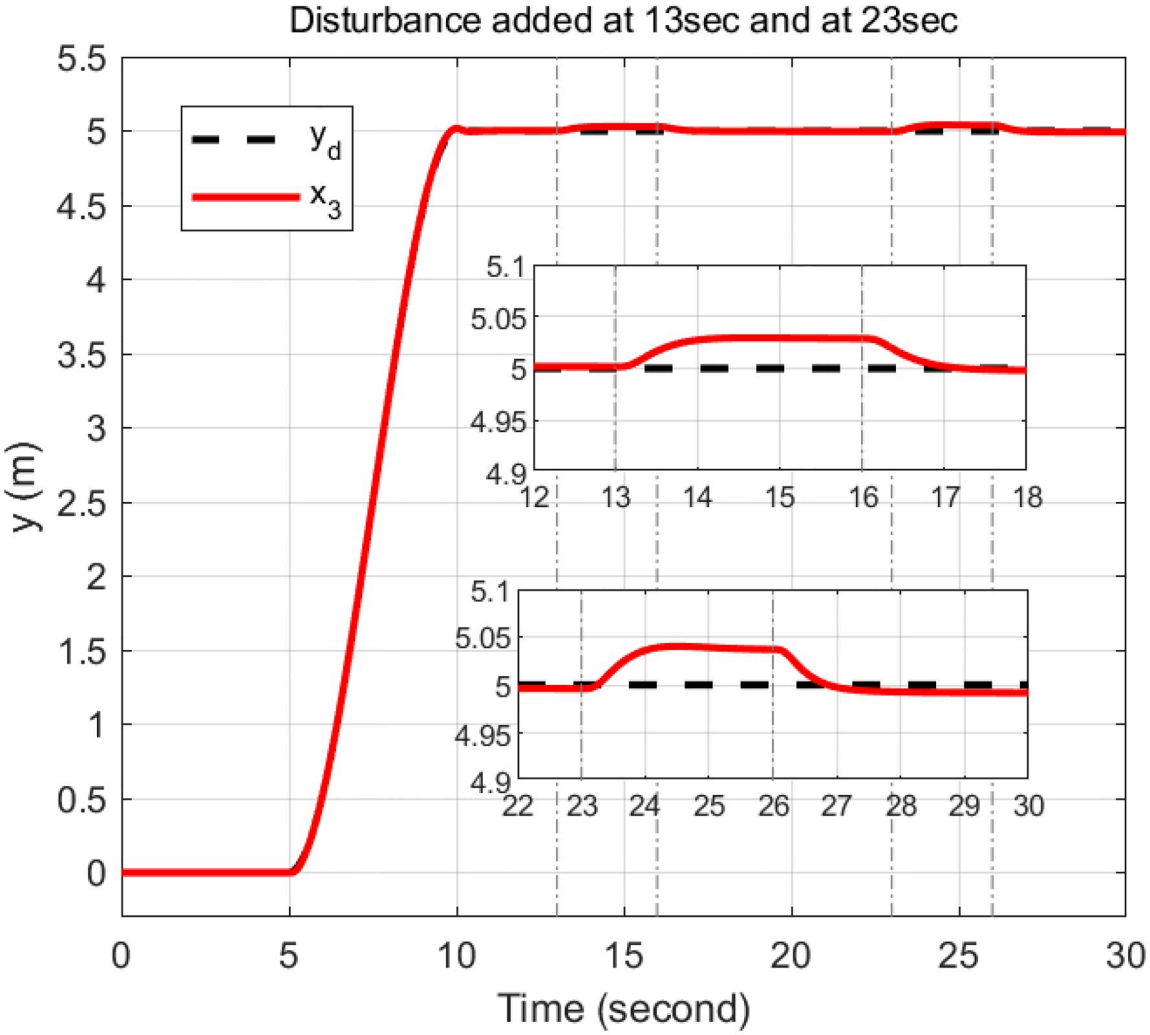
Disturbance rejection capacity of the proposed controller for the *y*-axis.

**Fig 22:**
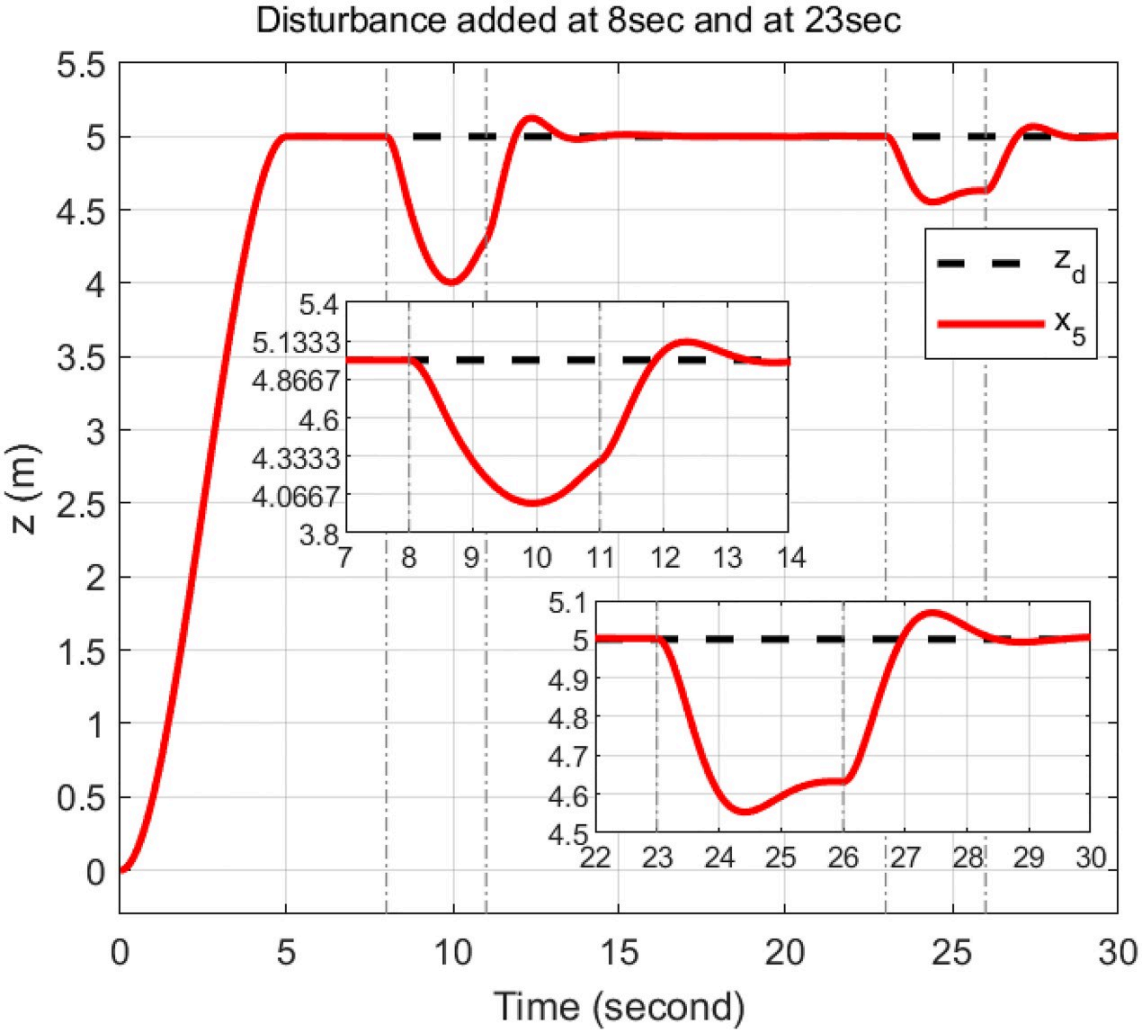
Disturbance rejection capacity of the proposed controller for the *z*-axis.

The response of the proposed controller to these disturbances demonstrates its exceptional efficiency and robustness. Despite the application of significant external forces and torques, the trajectory of the quadcopter is effectively maintained by the controller with minimal deviations. For *u*_1_, a disturbance applied from 8 to 11 seconds is rejected by the controller within 2.4 seconds after removal, while a subsequent disturbance from 23 to 26 seconds is rejected within 2.1 seconds. For *u*_2_, the disturbance from 13 to 16 seconds is mitigated within 0.9 seconds, and the disturbance from 23 to 26 seconds is resolved within 1 seconds. For *u*_3_, a disturbance applied from 18 to 21 seconds is managed within 1 seconds, and a disturbance from 23 to 26 seconds is managed within 2.2 seconds. These results illustrate the controller’s capability to handle multiple, sequential disturbances and to reject them promptly after removal, as depicted in Figs 20–22.

### 5.5 Parameter variation handling capacity

To demonstrate the efficiency and robustness of the proposed controller in tracking the desired trajectories under parameter variations, the following desired trajectories are used: *x*_*d*_ = 1 − cos(*t*), *y*_*d*_ = sin(*t*), and *z*_*d*_ = *t*.

The mass of the quadcopter is increased from 0.65 kg to 0.9 kg between 10 and 12 seconds, representing an addition of 0.25 kg. This increase corresponds to a 38.46% rise in the quadcopter’s mass. Consequently, the inertia constants *I*_*xx*_ and *I*_*yy*_ increase from 7.5 × 10^−3^ kg·m^2^ to 1.038 × 10^−2^ kg·m^2^, reflecting a 38.4% increase. Similarly, *I*_*zz*_ increases from 1.3×10^−2^ kg·m^2^ to 1.8 × 10^−2^ kg·m^2^, corresponding to a 38.46% increase. The inertia about the propeller axis *J* also increases from 6.0 × 10^−5^ kg·m^2^ to 8.31 × 10^−5^ kg·m^2^, which is a 38.5% increase.

**Fig 23:**
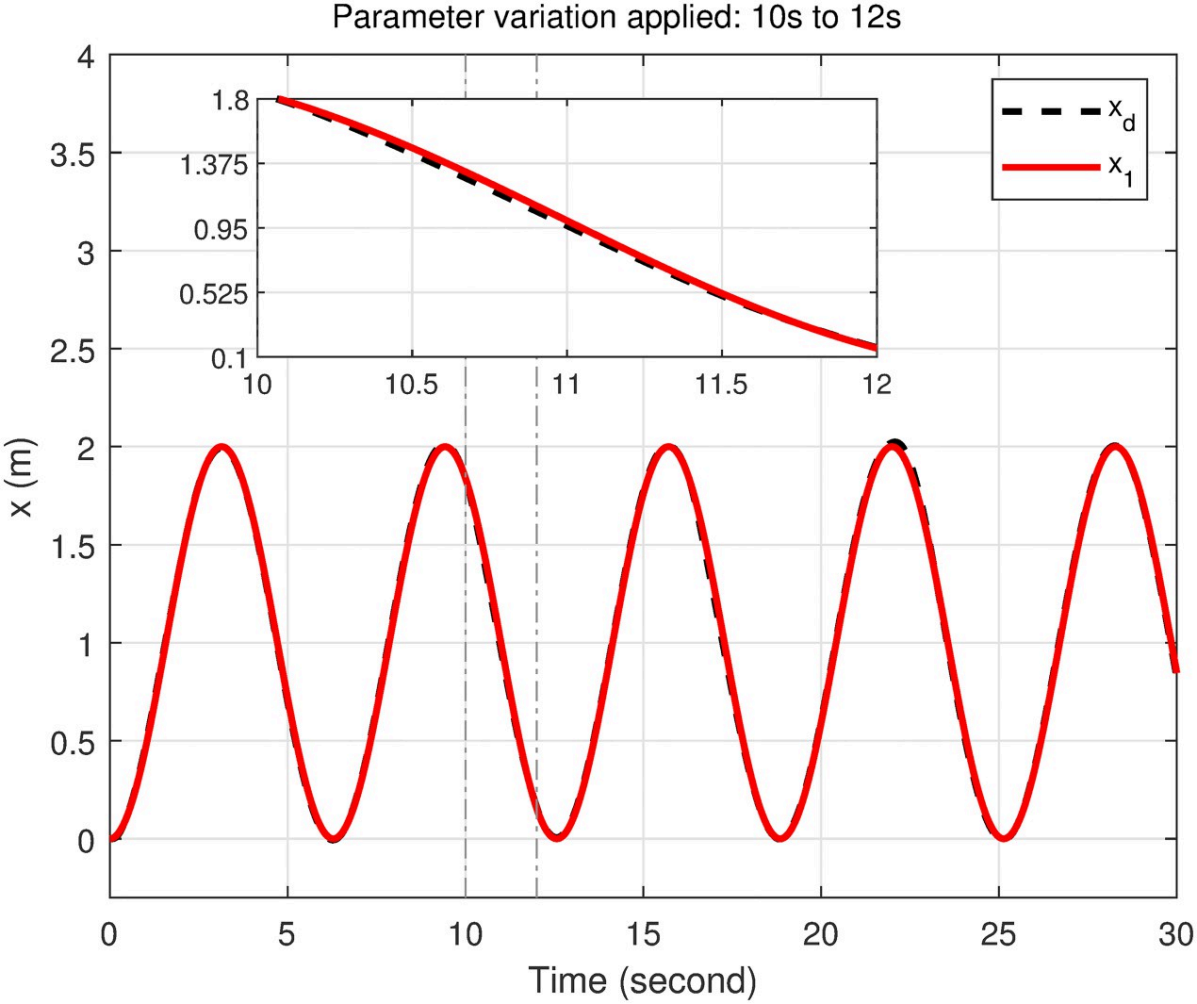
Parameter variation handling capacity of the proposed controller for the *x*-axis.

**Fig 24:**
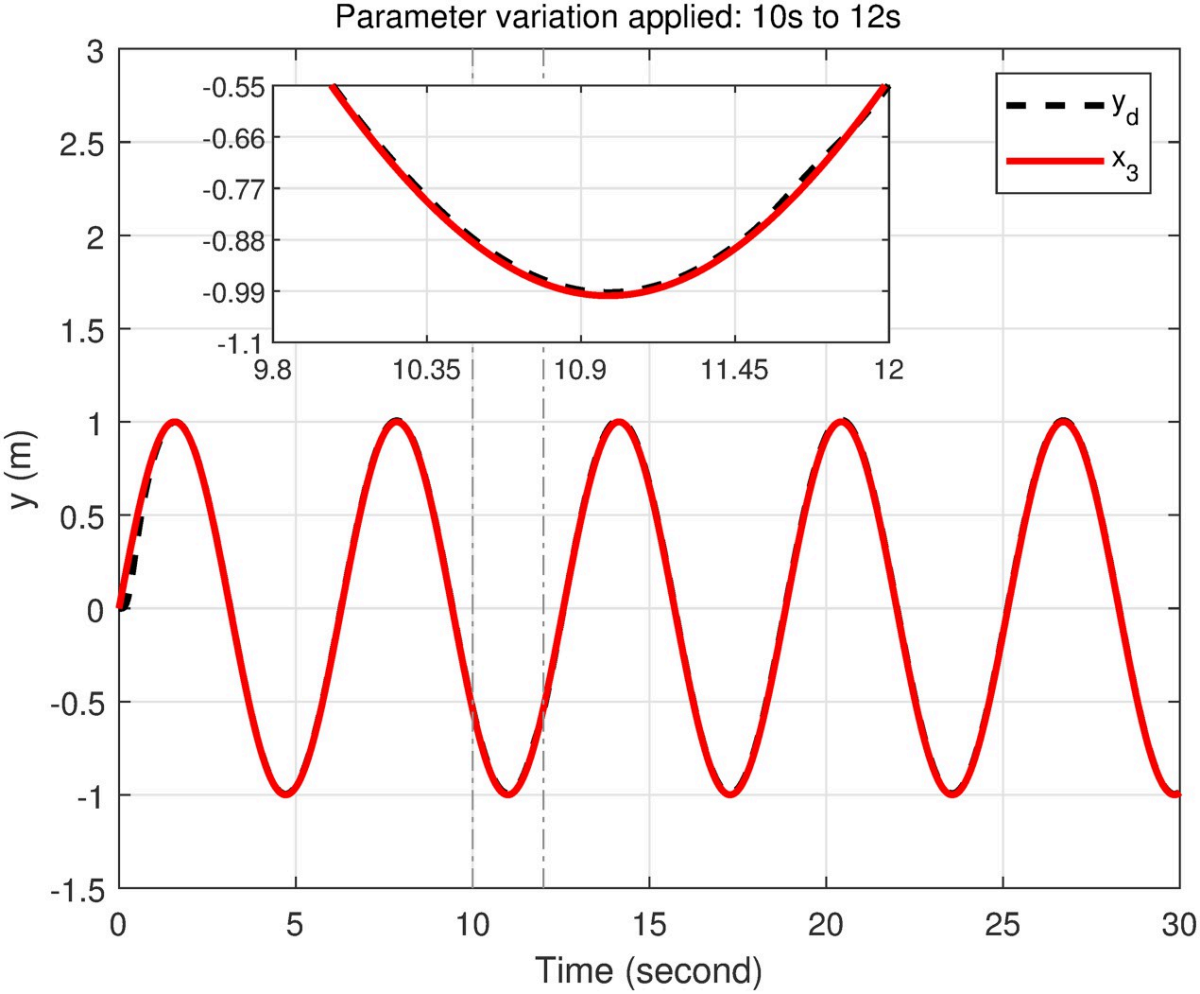
Parameter variation handling capacity of the proposed controller for the *y*-axis.

**Fig 25:**
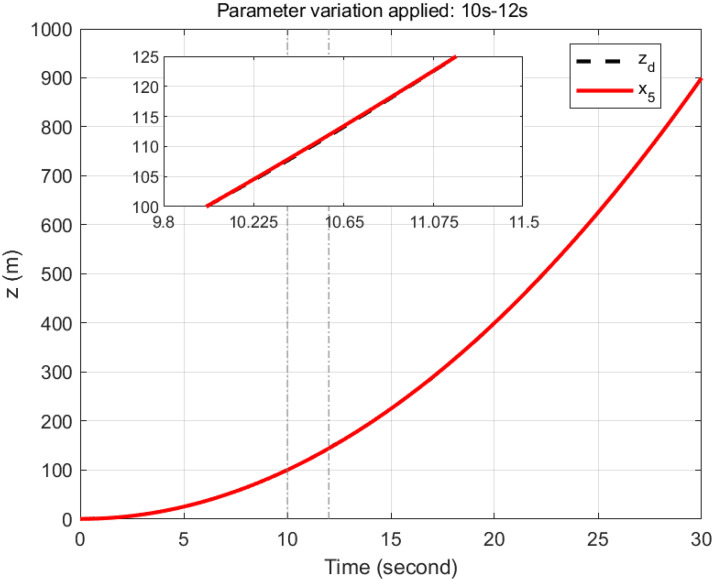
Parameter variation handling capacity of the proposed controller for the *z*-axis.

**Fig 26:**
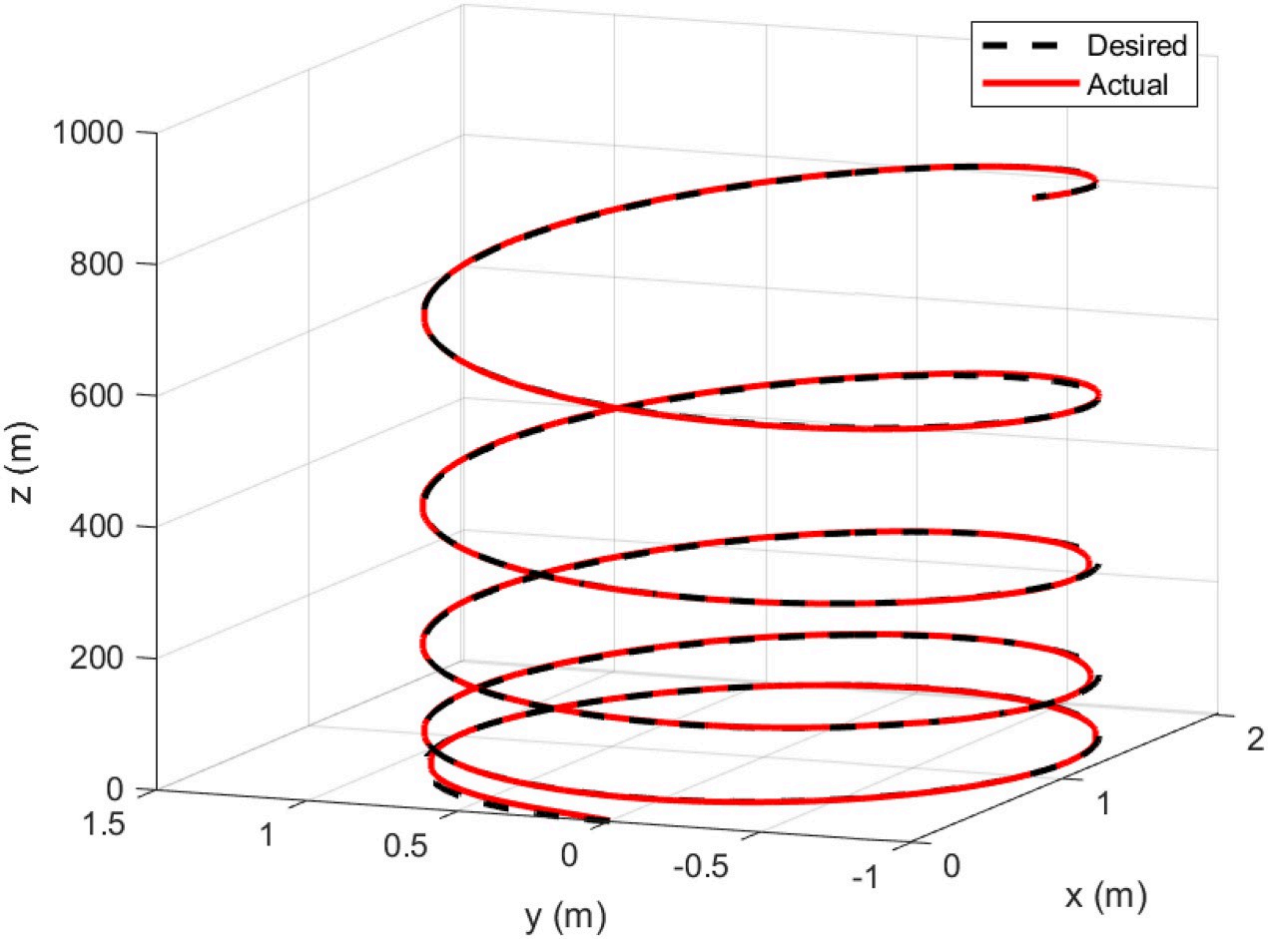
3D parameter variation handling capability of the proposed controller.

As seen in Figs 23–26 the proposed controller effectively handles the parameter variation in the mass of the quadcopter. It demonstrates robustness by maintaining accurate trajectory tracking despite the significant increase in mass. This highlights the controller’s ability to adapt to changing system dynamics and maintain stability and performance under varying conditions. Such robustness is crucial for real-world applications where system parameters are often subject to unpredictable changes.

### 5.6 Helical trajectory

A helical trajectory is generated through the specified desired trajectories: *x*_*d*_ = 1 − cos(*t*), *y*_*d*_ = sin(*t*), and *z*_*d*_ = *t*. A comparison is made between the proposed controller and other approaches.

**Fig 27:**
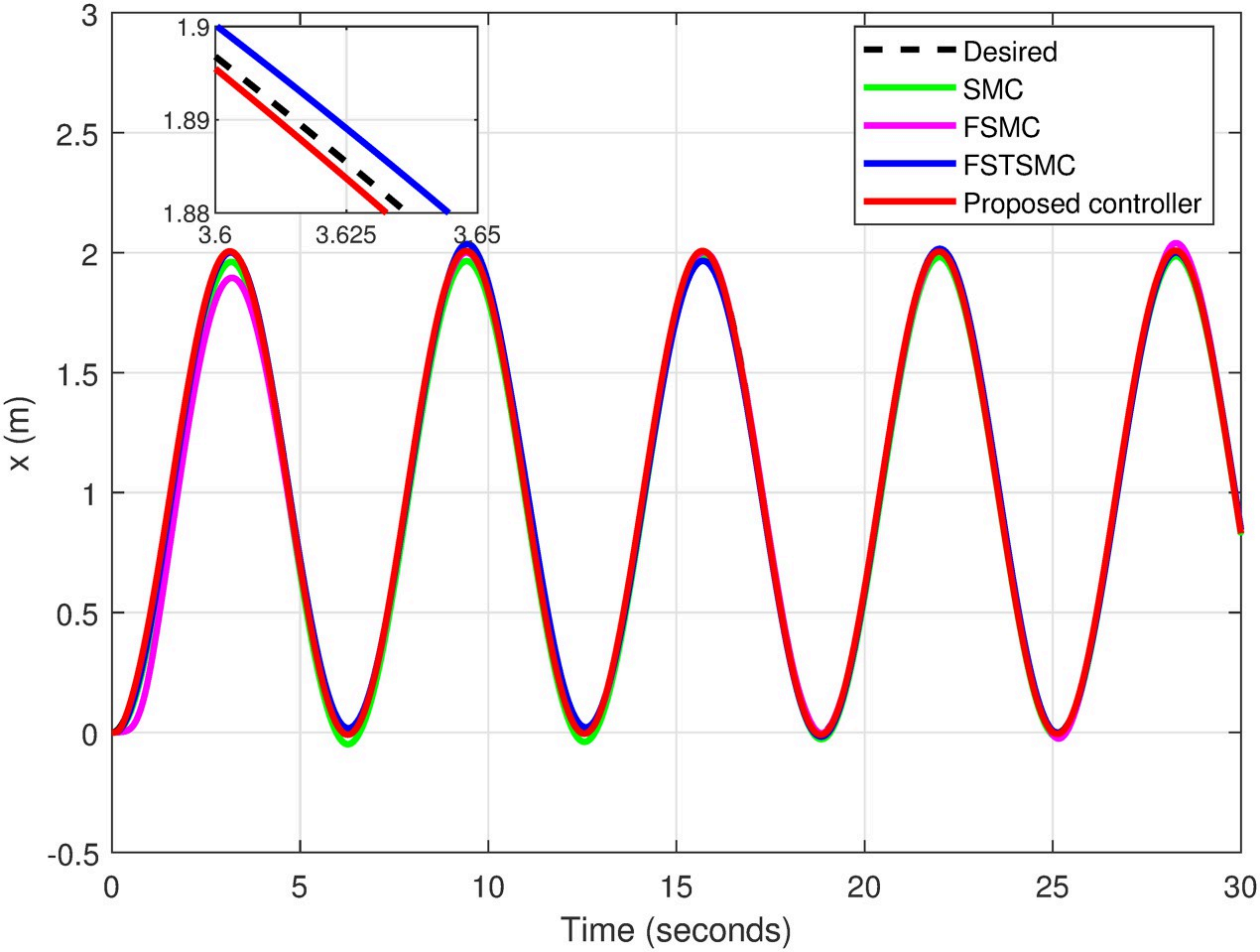
Tracking response for the *x*-axis.

**Fig 28:**
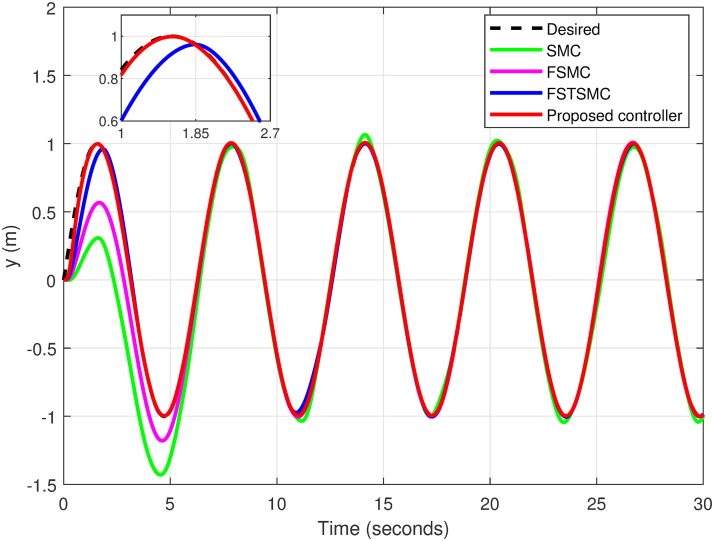
Tracking response for the *y*-axis.

**Fig 29:**
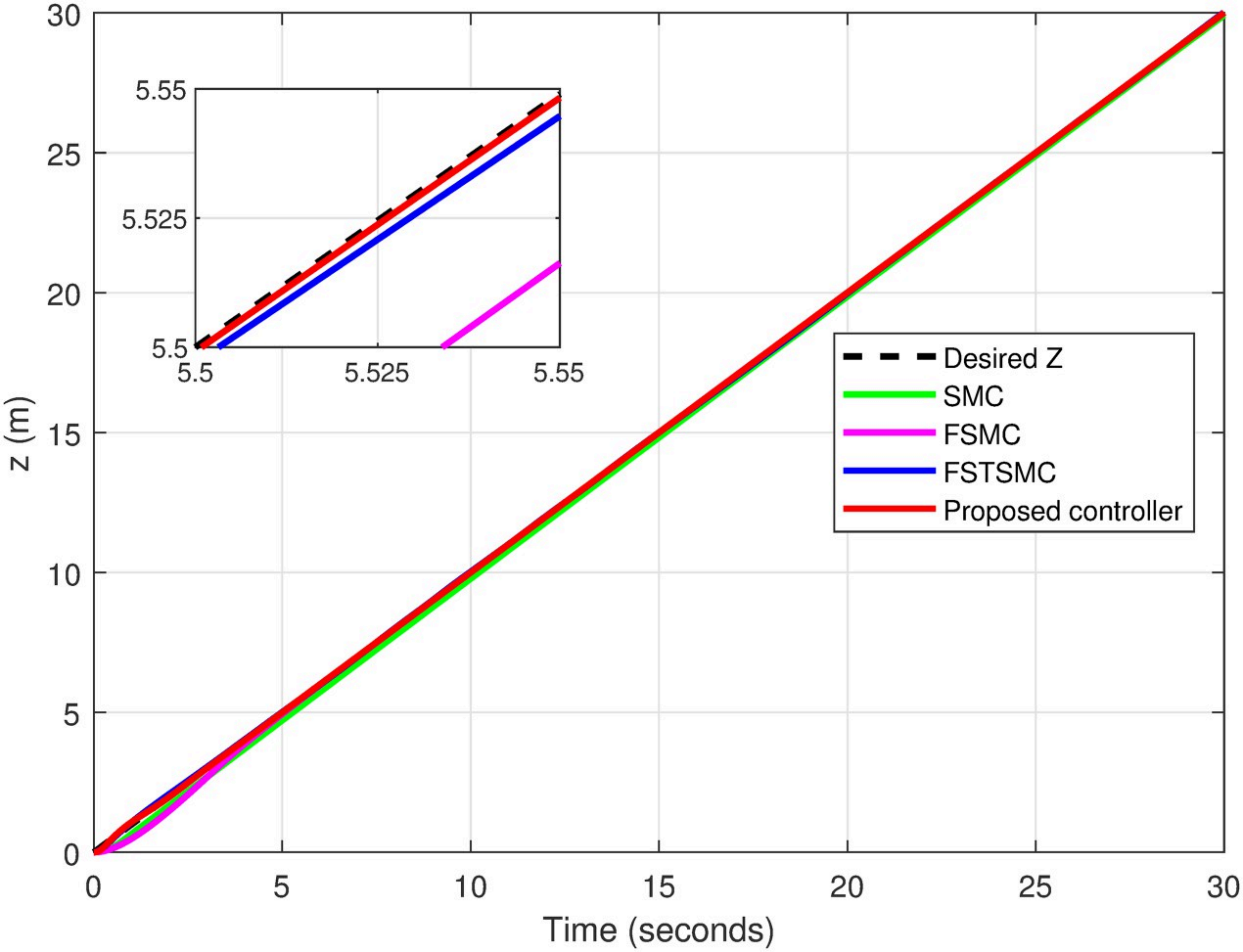
Tracking response for the *z*-axis.

**Fig 30:**
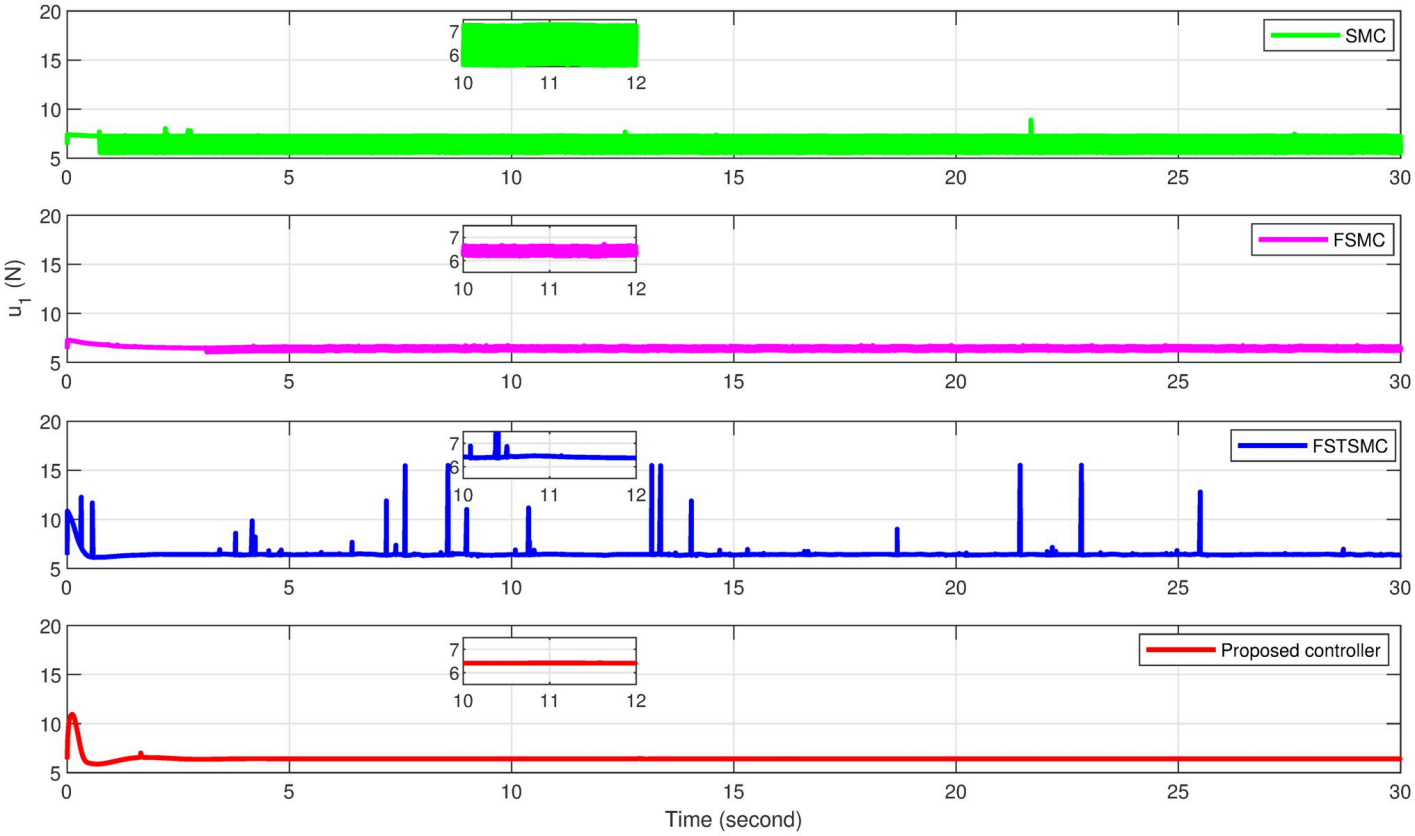
Altitude controller effort of quadcopter.

**Fig 31:**
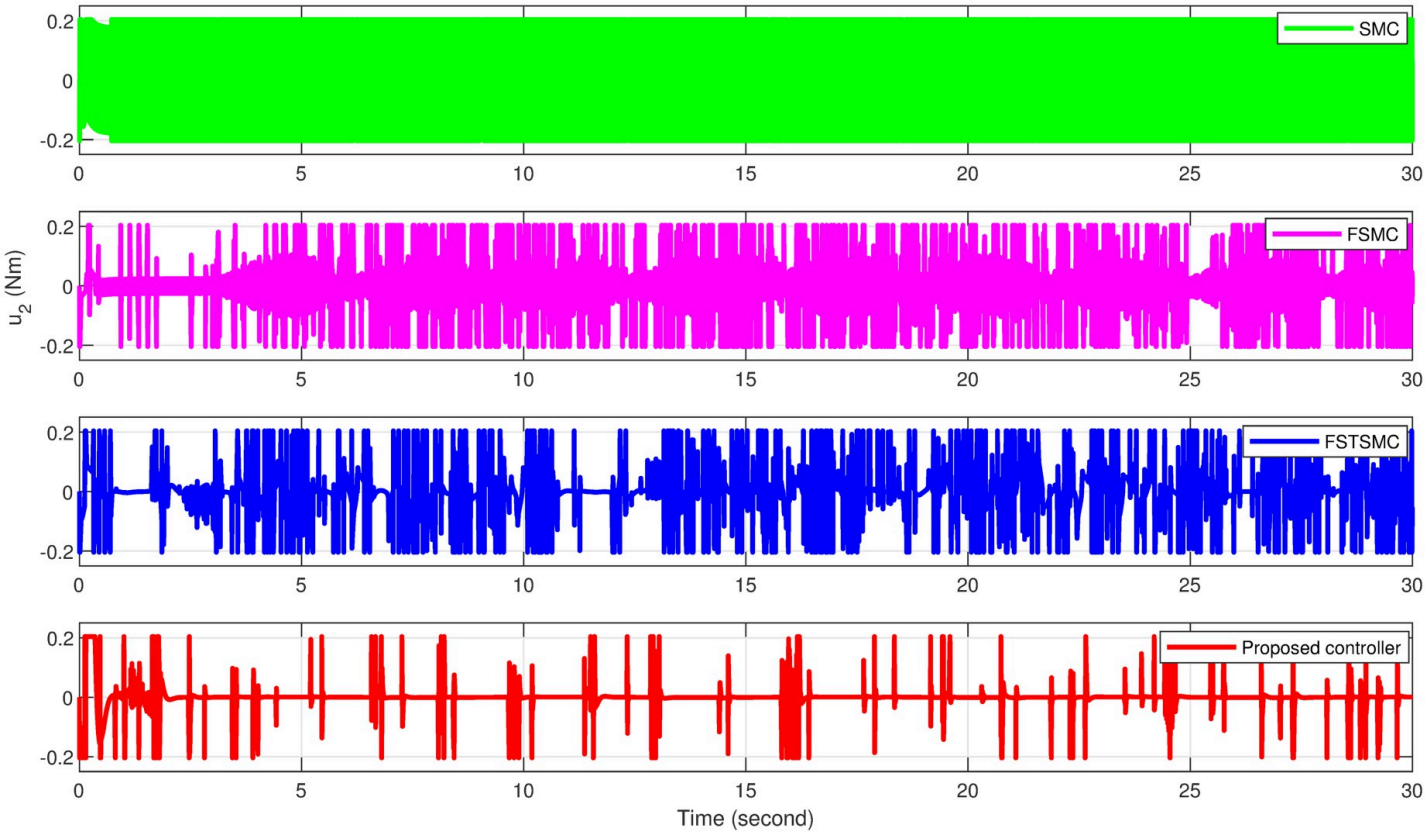
Attitude controller *u*_2_ effort for quadcopter.

**Fig 32:**
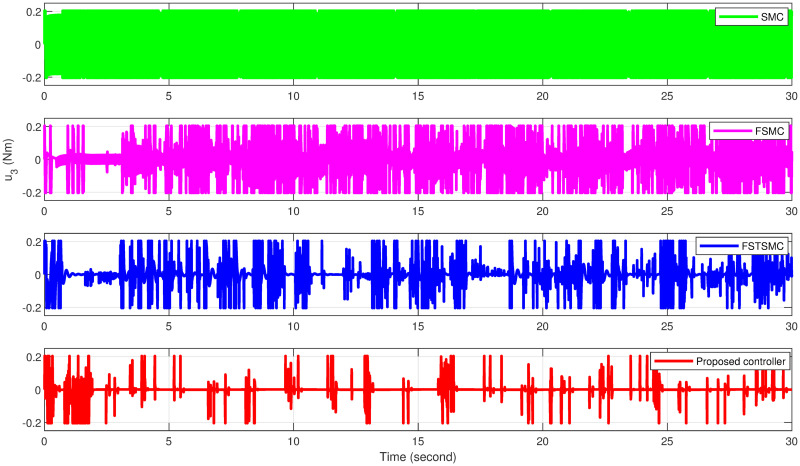
Attitude controller *u*_3_ effort for quadcopter.

The simulation results showcase the superior tracking performance of the proposed controller, as evidenced by its ability to quickly align the system states with the desired reference signals. Unlike the alternatives evaluated, which exhibit notably lower performance characterized by sluggish convergence rates, the proposed controller demonstrates a marked improvement in trajectory tracking. A particularly noteworthy advantage of the proposed solution is its effective reduction of control input chattering, a feature vividly illustrated in Figs 27–32. These figures provide a visual comparison that underscores the smoother control inputs achieved with the proposed method. This reduction in chattering not only signifies an improvement in control signal quality but also contributes to the longevity and reliability of the system components subjected to these inputs.

Table 4 shows the proposed controller exhibits reductions in error for variables *x*, *y*, and *z* by 63.45%, 94.41%, and 97.49%, correspondingly, in comparison to the errors observed with SMC. In contrast, reductions of 52.55%, 87.32%, and 91.54% for variables *x*, *y*, and *z*, respectively, are realized when comparing the proposed controller to FSMC. Additionally, when compared to FSTSMC, reductions of 28.57%, 61.77%, and 76.03% for variables *x*, *y*, and *z*, respectively, are achieved.

**Table 4:** IAE for *t* = 30 seconds.

	x	y	z
SMC	0.8837	3.858	5.571
FSMC	0.6807	1.7	1.652
FSTSMC	0.4522	0.5639	0.5827
Proposed controller	0.323	0.2156	0.1397

Table 5 compares the integral time error and highlights the superior performance of the proposed controller, with significantly lower errors. Table 6 presents the integral squared error, where the proposed controller consistently achieves the lowest errors across all metrics. Table 7 illustrates the integral time squared error, showcasing the proposed controller’s exceptional performance in reducing errors.

**Table 5:** ITE for *t* = 30 seconds.

	x	y	z
SMC	-10.54	-12.2	-64.42
FSMC	-0.3274	-3.526	-3.532
FSTSMC	-0.1636	0.3225	-2.538
Proposed controller	-0.0051	1.495	-0.2708

**Table 6:** ISE for *t* = 30 seconds.

	x	y	z
SMC	0.8837	1.851	1.216
FSMC	0.6807	0.5558	0.6626
FSTSMC	0.4522	0.0853	0.0247
Proposed controller	0.323	0.0145	0.0058

**Table 7:** ITSE for *t* = 30 seconds.

	x	y	z
SMC	4.231	27.55	153.1
FSMC	1.601	3.749	2.613
FSTSMC	2.182	0.706	3.091
Proposed controller	1.257	0.1248	0.0233

Table 8 illustrates that the proposed controller achieves reductions in control effort for variables *u*_1_, *u*_2_, and *u*_3_ by 0.052%, 94.06%, and 95.90%, respectively, compared to the effort observed with SMC. Conversely, reductions of 0%, 79.31%, and 89.30% for variables *u*_1_, *u*_2_, and *u*_3_, respectively, are observed when comparing the proposed controller to FSMC. Furthermore, when compared to FSTSMC, reductions of 0%, 67.56%, and 70.66% for variables *u*_1_, *u*_2_, and *u*_3_, respectively, are attained.

**Table 8:** Force and Torque for t = 30 seconds.

	*u*_1_ (N)	*u*_2_ (Nm)	*u*_3_ (Nm)
SMC	193.1	5.87	5.722
FSMC	193	1.684	2.189
FSTSMC	193	1.074	0.7983
Proposed controller	193	0.3484	0.2342

## 6 Conclusion

In this paper, modeling of quadcopter using the Newton Euler method has been developed. The fuzzy super twisting sliding mode control with fuzzy PID surface has been proposed as an inner and outer loop controller to manage the trajectory of a quadcopter. The integration of a fuzzy logic controller has proven to be a pivotal solution to the inherent complexity of tuning STSMC parameters with PID sliding surfaces. By incorporating the super twisting algorithm, the system successfully reduced the chattering problem. Furthermore, the automatic tuning of STSMC parameters considerably lightened the computational load of the control, while the addition of an integral term sliding surface substantially reduced steady-state errors. Rigorous testing under various conditions, including different trajectories and disturbances, demonstrated the exceptional control performance. This system adeptly tracked desired trajectories, rejected disturbances, and maintained stability. The fusion of Fuzzy logic and STSMC with PID sliding surfaces has enhanced robustness, reduced chattering, and accelerated state convergence while maintaining stability. However, the desired trajectory waypoints are generated manually. This desired trajectory can be generated automatically using path planning which will be considered in the future work.

## Supporting information

S1 Appendix Proof of stability analysis for SMC with PID surface. (PDF)

S2 Appendix Proof of stability analysis for STSMC with PID surface. (PDF)
