# Supplementary material for: An improved trajectory tracking control of quadcopter using a novel Sliding Mode Control with Fuzzy PID Surface
Source: PLoS One. 2024 Nov 21;19(11):e0308997. doi: 10.1371/journal.pone.0308997 (PMC11581241; doi:10.1371/journal.pone.0308997)
Supplement: S1 Appendix — (PDF) [file pone.0308997.s001.pdf]

S1 Appendix: Proof of stability analysis for SMC with PID surface

Defining Lyapunov candidate for x-subsystem

$$V(s_1) = \frac{1}{2}s_1^2 \tag{S1.1}$$

Since $V(0) = 0$ and $V(s_1) > 0$, it is positive definite. Taking the derivative

$$\dot{V}(s_1) = s_1\dot{s}_1 \tag{S1.2}$$

Substituting the time derivative of the sliding surface and rearranging the equation becomes

$$\dot{V}(s_1) = -s_1 Q_x \text{sign}(s_1) \tag{S1.3}$$

If $s_1 > 0$, $Q_x > 0$ and if $s_1 < 0$, $Q_x > 0$, so Q_x must be positive to be stable. The same is true for y, z, ϕ, θ, ψ. This completes the proof.
