# Supplementary material for: An improved trajectory tracking control of quadcopter using a novel Sliding Mode Control with Fuzzy PID Surface
Source: PLoS One. 2024 Nov 21;19(11):e0308997. doi: 10.1371/journal.pone.0308997 (PMC11581241; doi:10.1371/journal.pone.0308997)
Supplement: S2 Appendix — (PDF) [file pone.0308997.s002.pdf]

S2 Appendix: Proof of stability analysis for STSMC with PID surface

Defining Lyapunov candidate for x-subsystem

$$V(s_1) = \frac{1}{2}s_1^2 \tag{S2.1}$$

Since $V(0) = 0$ and $V(s_1) > 0$, it is positive definite. Taking the derivative

$$\dot{V}(s_1) = s_1\dot{s}_1 \tag{S2.2}$$

Substituting the time derivative of the sliding surface and rearranging the equation becomes

$$\dot{V}(s_1) = s_1\left(-k_1|s_1|^{1/2}\mathrm{sign}(s_1) - k_2\int \mathrm{sign}(s_1)\,dt\right) \tag{S2.3}$$

If $s_1 > 0$, to make $\dot{V}(s_1) < 0$, k_1 and k_2 must be positive. If $s_1 < 0$, to make $\dot{V}(s_1) < 0$, k_1 and k_2 must be positive. The same analysis applies for y, z, ϕ, θ, ψ.
